# GRMT: Generative Reconstruction of Mutation Tree From Scratch Using Single-Cell Sequencing Data

**DOI:** 10.3389/fgene.2021.692964

**Published:** 2021-06-04

**Authors:** Zhenhua Yu, Huidong Liu, Fang Du, Xiaofen Tang

**Affiliations:** ^1^School of Information Engineering, Ningxia University, Yinchuan, China; ^2^Collaborative Innovation Center for Ningxia Big Data and Artificial Intelligence Co-founded by Ningxia Municipality and Ministry of Education, Ningxia University, Yinchuan, China

**Keywords:** next-generation sequencing, single-cell sequencing, Bayesian optimization, intra-tumor heterogeneity, tumor tree

## Abstract

Single-cell sequencing (SCS) now promises the landscape of genetic diversity at single cell level, and is particularly useful to reconstruct the evolutionary history of tumor. There are multiple types of noise that make the SCS data notoriously error-prone, and significantly complicate tumor tree reconstruction. Existing methods for tumor phylogeny estimation suffer from either high computational intensity or low-resolution indication of clonal architecture, giving a necessity of developing new methods for efficient and accurate reconstruction of tumor trees. We introduce GRMT (Generative Reconstruction of Mutation Tree from scratch), a method for inferring tumor mutation tree from SCS data. GRMT exploits the *k*-Dollo parsimony model to allow each mutation to be gained once and lost at most *k* times. Under this constraint on mutation evolution, GRMT searches for mutation tree structures from a perspective of tree generation from scratch, and implements it to an iterative process that gradually increases the tree size by introducing a new mutation per time until a complete tree structure that contains all mutations is obtained. This enables GRMT to efficiently recover the chronological order of mutations and scale well to large datasets. Extensive evaluations on simulated and real datasets suggest GRMT outperforms the state-of-the-arts in multiple performance metrics. The GRMT software is freely available at https://github.com/qasimyu/grmt.

## 1. Introduction

Tumor progression follows a dynamic evolutionary process that is activated by the genetic lesions of a single founder cell (Nowell, 1976). The descendants of the cell gain a growth advantage to resist apoptosis and develop into subclones through accumulation of somatic mutations. After many generations of clonal expansion, distinct cell populations emerge in the tumor and relate with an evolutionary tree that depicts their chronological relationship. Each cell population constitutes a subclone that is uniquely characterized by a complement of genetic mutations. The genetic diversity of the subclones is called as the intra-tumor heterogeneity (Nowell, 1976; Greaves and Maley, 2012; Swanton, 2012), and provides the cues of key mutations that drive tumor growth. Therefore, accurately disentangling the clonal composition and underlying evolutionary relationship is essential for finding the driver mutations (Xi et al., 2018, 2020) that dominate the tumor progression, and helps design of personalized cancer therapies (Stratton et al., 2009; Swanton, 2012).

With the breakthrough of whole-genome amplification (WGA) (Zong et al., 2012) and single cell isolation (Brasko et al., 2018) technologies, single-cell DNA sequencing (SCS) (Gawad et al., 2016) now promises a high-resolution landscape of the genetic diversity at single cell level. SCS allows for the reconstruction of tumor evolutionary tree by exploiting the mutation profiles of single cells. However, the current WGA procedures in SCS inevitably introduce different types of noise that contribute considerably to the genotyping errors, making the data obtained from SCS experiments notoriously error-prone. Allele dropout (ADO) is a prominent technical issue that results in false negative (FN) errors in SCS data (Navin, 2014). The reported FN rates in current SCS-based studies vary from 0.1 to 0.43 (Hou et al., 2012; Xu et al., 2012; Gawad et al., 2014; Wang et al., 2014). False positive (FP) calls also present with an elevated rate compared to bulk-sequencing. A consensus based trick is often employed to attenuate the effect of FP errors by filtering the mutations only observed in one single cell (Zhang et al., 2015; Zafar et al., 2016), however this approach may result in removal of true biological mutations unique to single cell. Unobserved sites can also be a critical issue caused by ADO and non-uniform coverage. The missing rate may exceed 50% due to the low quality of sequencing data (Hou et al., 2012). Lastly, cell doublets act as another type of noise in SCS data that result from unintended libraries from two or more cells (Roth et al., 2016; Zafar et al., 2017). The cell doublet rate can be as high as 10% in oral pipette and droplet encapsulation cell isolation techniques (Hou et al., 2012; Xu et al., 2012; Macosko et al., 2015). Critically, aforementioned issues often occur together in SCS data, making it much complicated to accurately infer the evolutionary tree.

There has been a great interest of developing computational tools for reasoning tumor trees by addressing the aforementioned issues in SCS data (Jahn et al., 2016; Kuipers et al., 2017; Zafar et al., 2017, 2019; El-Kebir, 2018; Chen et al., 2020; Myers et al., 2020; Sadeqi Azer et al., 2020). SCITE (Jahn et al., 2016) exploits a Markov Chain Monte Carlo (MCMC) based approach to jointly search the best scoring mutation tree and FN rate. OncoNEM (Ross and Markowetz, 2016) adopts a heuristic search algorithm to find best-performing subclonal tree refined by unobserved clones. SCG (Roth et al., 2016) depends on a hierarchical Bayesian model to group single cells into subclones. These methods are built by following the infinite sites model (ISM) that each site gets mutated only once and the mutation will not be lost once acquired. The assumption may often not hold for human tumor evolution where loss of mutations frequently occurs due to copy number alterations (CNAs). To relax the constraint, SiFit (Zafar et al., 2017) adopts the finite site model (FSM) to permit back mutation and parallel evolution, and infers a maximum likelihood estimation of the cell lineage tree. Another method called BEAM (Miura et al., 2018) aims to improve the quality of SCS data using classical molecular evolutionary phylogenetics without explicit restrictions on evolutionary model. SPhyR (El-Kebir, 2018) infers tumor phylogeny based on the Dollo parsimony evolutionary model (Dollo, 1893) that is slightly more restrictive than FSM and only allows back mutation. As loss of mutations is the main factor that contributes to homoplasy in tumor evolution, the Dollo parsimony model is a good tradeoff between ISM and FSM. Recently, RobustClone (Chen et al., 2020) is proposed to efficiently reconstruct subclonal evolution tree via robust principal component analysis. Several methods additionally incorporate other information to improve tumor tree inference (Satas et al., 2020; Wu, 2020). For instance, ScisTree (Wu, 2020) utilizes genotype uncertainty available from the results of genotype callers to model non-uniformly distributed errors in genotypes.

While the existing methods perform acceptably well, there are certain drawbacks that limit their applications. First, MCMC based methods like SCITE and SiFit suffer from high computational intensity when applied to large datasets (Chen et al., 2020). Second, methods that search for subclonal trees (e.g., SPhyR and RobustClone) may fail to detect low-prevalence subclones and thus result in an incomplete and low-resolution indication of clonal architecture. Third, most of the existing methods are based on ISM that is not in line with the underlying tumor evolution where loss of mutations occurs frequently. Finally, to the best of our knowledge, mutation tree that represents the highest-resolution indication of tumor evolutionary process is reported by only one method SCITE. As SCITE does not scale well on large datasets, methods for efficient and accurate reconstruction of mutation tree are still highly needed.

In this study, we introduce a novel method called GRMT for Generative Reconstruction of Mutation Tree from scratch using SCS data. GRMT employs the *k*-Dollo parsimony model to add a constraint on mutation evolution, i.e., each mutation can be gained once and lost at most *k* times. Unlike previous approaches that yield different tree topologies via structure transformation (e.g., swap of nodes or subtrees), GRMT searches for mutation tree structures from a perspective of tree generation from scratch. Formally, reconstruction of mutation tree is depicted as a generative process that begins with the initial tree that only encompass root node, proceeds with attachment of a new node per time that represents gain or loss of a mutation to the tree, and terminates with the complete structure that contains all mutations. To prevent overfitting, we define a score metric to evaluate the goodness of each tree, and early stopping of tree generation is activated when the monitored metric is less than a pre-defined threshold. In GRMT framework, chronological order of mutations is expressed by a tree growing process, therefore the proposed generative model is intuitively more suitable for deciphering the evolutionary history of the tumor. In addition, we employ Bayesian optimization (BO) algorithm to efficiently infer the error rates in SCS data. We apply GRMT to various simulated datasets to show its superior performance in mutation tree inference, and also demonstrate the effectiveness of GRMT in real data.

## 2. Materials and Methods

**Figure 1:**
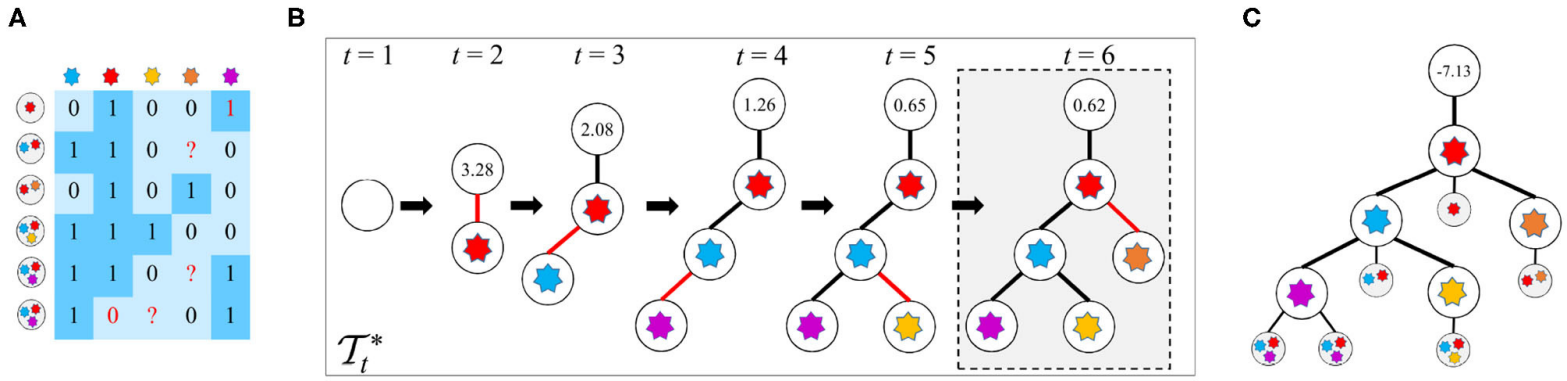
An illustration of the mutation tree reconstruction procedure adopted in GRMT. Our method generatively recover the mutation tree from a perspective of tree generation from scratch based on the *k*-Dollo parsimony model. **(A)** An example of the observed mutation data with 6 cells and 5 mutations denoted by a 6×5 matrix, the elements 1 and 0 marked in red color represent false positive and false negative, respectively, and the symbol “?” means missing data. **(B)** GRMT generatively rebuilds the mutation tree by iteratively increasing the tree size. Parameters α, β, *k*, λ, and κ are set to 0.077, 0.071, 0, 0.7, and 0.5, respectively. The value contained in each root node denotes $s\left(T_{t}^{*}\right)$ of the best tree $T_{t}^{*}$ at time *t*. The tree encompassed by dotted lines is the optimal solution found by our method. **(C)** The cells are attached to mutation tree by maximizing likelihoods, and the value contained in root node of the mutation tree represents the log-likelihood of the observed mutation data.

Given the observed mutation data *D*, the FP rate (FPR) α and FN rate (FNR) β in *D*, and the parameter *k* of the *k*-Dollo parsimony model, the workflow of reasoning optimal mutation tree is as follows: (1) for each of the *M* mutations, generate *k* + 1 nodes of which one represents gain of the mutation and the rest denote loss of the mutation, yielding in total (*k* + 1)*M* isolated nodes as well as a root node indicating no mutations; (2) initialize the tree $T_{1}^{*}$ to only contain the root node; (3) generate new trees $\left\{T_{t+1}\right\}$ by connecting a free node to the previous tree $T_{t}^{*}$; (4) evaluate all proposals in step 3 to keep the best tree $T_{t+1}^{*}$; (5) iteratively repeat steps 3 and 4 until there are no free nodes or the score of $T_{t+1}^{*}$ is less than a predefined threshold. During the whole process, a globally optimal tree $T^{*}$ is updated when a new better tree is found. The cells are then attached to $T^{*}$ via maximizing likelihoods. An illustration of the mutation tree inference procedure is shown in Figure 1. The tree encompassed by dotted lines is the best solution found by our method. The following sections give a description of methodological details of GRMT.

### 2.1. Formulating Mutation Data

We present the mutation states of *N* cells at *M* genomic loci as a *N* × *M* binary matrix *E*, where *E*_*ij*_ = 1 and *E*_*ij*_ = 0 denote the presence and absence of mutation *j* in cell *i*, respectively. The observed mutation data *D* is a noisy version of *E*, and the probability distribution of *D*_*ij*_ can be formulated as:

(1) $$\begin{array}{l} p\left(D_{ij}|E_{ij}\right)=\left(\begin{array}{cc} p\left(0|0\right) & p\left(1|0\right)\\ p\left(0|1\right) & p\left(1|1\right) \end{array}\right)=\left(\begin{array}{cc} 1-\alpha & \alpha \\ \beta & 1-\beta \end{array}\right) \end{array}$$

For ternary mutation matrix *D* whose elements take value from {0, 1, 2}, where 0 denotes normal state, 1 denotes heterozygous mutation, and 2 means that a heterozygous mutation is recorded as homozygous due to allele dropout, we use the similar probability distribution of *D*_*ij*_ as adopted in Jahn et al. (2016):

(2) $$\begin{array}{l} \begin{array}{c} p\left(D_{ij}|E_{ij}\right)=\left(\begin{array}{ccc} p\left(0|0\right) & p\left(1|0\right) & p\left(2|0\right)\\ p\left(0|1\right) & p\left(1|1\right) & p\left(2|1\right) \end{array}\right)\\ \;=\left(\begin{array}{ccc} 1-\alpha -\alpha \beta /2 & \alpha & \alpha \beta /2\\ \beta /2 & 1-\beta & \beta /2 \end{array}\right) \end{array} \end{array}$$

Given a mutation tree, each cell is attached to the internal node of the tree that maximizes the likelihood of the observed mutation data. Provided that the *i*-th cell is derived from the *n*-th internal node, the likelihood can be calculated as $p\left(D_{i}|S_{n}\right)=\prod_{j=1}^{M}p\left(D_{ij}|S_{nj}\right)$. Here *S*_*n*_ is a vector with length of *M* and denotes the underlying mutation state associated with the *n*-th internal node. The value of *S*_*n*_ can be deduced by traversing the path from the root to the *n*-th internal node that gives the evolution history of mutations along that path. If loss of mutation occurs after a mutation is gained, the corresponding element of *S*_*n*_ is set to 0. Suppose the best locations of attachments for all cells are represented by ξ = (ξ_1_, ξ_2_, ..., ξ_*N*_) under a tree T, then the log-likelihood is given by:

(3) $$\begin{array}{l} l\left(D|T,\alpha ,\beta \right)=\overset{N}{\underset{i=1}{\sum }}\log\left(p\left(D_{i}|S_{\xi_{i}}\right)\right) \end{array}$$

### 2.2. Constructing Mutation Tree

Given FPR α and FNR β, we aim to find the tree $T^{*}$ that best explains the observed mutation data. To explicitly model the loss of mutations due to copy number alterations and loss of heterozygosity that are frequently observed in cancer genomes, the *k*-Dollo parsimony model is employed in GRMT to add a constraint on mutation evolution, i.e., each mutation can be gained once and lost at most *k* times. Formally, the internal nodes of the mutation tree is denoted by a vector S=(r,a+,a-,a-,…,a-,b+,b-,b-,…), where *r* signifies the root of the tree, *a*+ represents gain of mutation *a*, and *a*− suggests loss of mutation *a* and appears *k* times in the vector. In addition, the structure of the mutation tree is depicted by a vector T of the same length to S, and each element of T indicates the index of parent of the corresponding node in S (we use 0 to denote root node and –1 to indicate no parent). The internal nodes of T are denoted by V = {v|T(v)≠-1}.

We start with $T_{1}^{*}=\left(0,-1,-1,\ldots ,-1\right)$ that only contains the root node, and iteratively increase the size of the tree through introduction of a new node per time. Specifically, neighborhoods of the precursor tree $T_{t}^{*}$ are generated as a set of trees $\left\{T_{t+1}\right\}$ where each $T_{p}^{c}\in \left\{T_{t+1}\right\}$ contains *t*+1 nodes and derives from connecting node *c* to the split point *p* (internal node) of $T_{t}^{*}$, by following the restriction that the node symbolized by *a*− can only be connected to an internal node of $T_{t}^{*}$ where mutation *a* has been gained and not yet lost. This results in at most *t*(*Mk* + *M* + 1 − *t*) neighborhoods. To find the most probable tree from all proposals in $\left\{T_{t+1}\right\}$, we propose a metric to score each $T_{p}^{c}$.

With an assumption of uniform prior probability distribution of cell attachment points, the posterior probability that the *i*-th cell derives from the node *p* of $T_{t}^{*}$ is measured as:

(4) $$\begin{array}{l} p\left(\xi_{i}=p|D_{i},T_{t}^{*}\right)=\frac{p\left(D_{i}|\xi_{i}=p,T_{t}^{*}\right)}{\underset{v\in V_{t}}{\sum }p\left(D_{i}|\xi_{i}=v,T_{t}^{*}\right)} \end{array}$$

where *V*_*t*_ denotes the internal nodes of $T_{t}^{*}$. We then calculate the expected number of cells attached to node *p* as:

(5) $$\begin{array}{l} \pi_{p}\left(T_{t}^{*}\right)=\overset{N}{\underset{i=1}{\sum }}p\left(\xi_{i}=p|D_{i},T_{t}^{*}\right) \end{array}$$

Note that, the tree $T_{p}^{c}$ is generated by adding edge < *p, c*> to $T_{t}^{*}$, the expected number of cells transferring from node *p* to node *c* can be measured as:

(6) $$\begin{array}{l} {\bar{\pi }}_{p}=\pi_{p}\left(T_{t}^{*}\right)-\pi_{p}\left(T_{p}^{c}\right) \end{array}$$

Similarlly, the expected number of cells that are originally located in nodes {*v*|*v* ∈ *V*_*t*_, *v* ≠ *p*} of $T_{t}^{*}$ and now attached to node *c* of $T_{p}^{c}$ is formulated as:

(7) $$\begin{array}{l} {\tilde{\pi }}_{p}=\pi_{c}\left(T_{p}^{c}\right)-{\bar{\pi }}_{p} \end{array}$$

Based on above definitions, we propose a score metric to compare different trees in $\left\{T_{t+1}\right\}$:

(8) $$\begin{array}{l} s\left(T_{p}^{c}\right)=\lambda {\bar{\pi }}_{p}+\left(1-\lambda \right){\tilde{\pi }}_{p} \end{array}$$

where λ is a hyper-parameter controlling the weights of the two terms. Conceptually, ${\bar{\pi }}_{p}$ is the direct “cell flow” from node *p* to *c*, and ${\tilde{\pi }}_{p}$ is the “cell flow” from other nodes to *c* via *p*. We give higher weight (λ > 0.5) to the term ${\bar{\pi }}_{p}$ to encourage tree expansion toward the direction of larger direct “cell flow.” Finally, the best tree at time *t*+1 is inferred as:

(9) $$\begin{array}{l} T_{t+1}^{*}=\underset{T_{t+1}}{\arg\;\max}\;s(T_{t+1}) \end{array}$$

A globally best tree $T^{*}$ is maintained and updated when a new better tree is found, i.e., $l\left(D|T_{t+1}^{*},\alpha ,\beta \right)>l\left(D|T^{*},\alpha ,\beta \right)$. During each iteration, only the probability $p\left(\xi_{i}=c|D_{i},T_{p}^{c}\right)$ needs to be calculated for each cell, therefore the mutation tree can be reconstructed with high efficiency. The tree grows until no free nodes are available or $s\left(T_{t+1}^{*}\right)$ is less than a predefined threshold κ. A brief illustration of the mutation tree inference procedure is provided in Algorithm 1.

### 2.3. Inferring the Error Rates

We formulate finding optimal α and β as the following optimization problem:

(10) $$\begin{array}{l} \left(\alpha^{*},\beta^{*})=\underset{\alpha ,\beta }{\arg\;\max}\;f(\alpha ,\beta \right) \end{array}$$

where $f\left(\alpha ,\beta \right)=l\left(D|T^{*},\alpha ,\beta \right)$ represents the log-likelihood associated with the best tree $T^{*}$ given parameters *x* = (α, β). As the computational complexity of assessing the value of *f*(*x*) is exponentially increased with the data size, the values of *x* should be judiciously selected for evaluation to reduce the computational cost. To achieve this, we propose an approach to generate a priority ordering of the values of *x* to evaluate by developing a search algorithm based on the BO. Formally, we place a Gaussian process (GP) prior on *f*(*x*) to infer it's posterior probability distribution at a candidate point *x*. To find the solution, we first sample *t* candidate points according to an initial space-filling design, and evaluate all points to obtain the values *f*(*x*_1:*t*_) = [*f*(*x*_1_), *f*(*x*_2_), …, *f*(*x*_*t*_)]. Leveraging the *t* evaluated points up to now, we compute the posterior probability distribution on *f* as follows:

(11) $$\begin{array}{l} f\left(x\right)|f\left(x_{1:t}\right)\sim N\left(\mu_{t}\left(x\right),\sigma_{t}^{2}\left(x\right)\right) \end{array}$$

where the posterior mean μ_*t*_(*x*) and posterior variance $\sigma_{t}^{2}\left(x\right)$ can be computed efficiently using the GP prior (Rasmussen and Williams, 2005).

**Algorithm 1:** Algorithm for mutation tree reconstruction. *D* is a mutation matrix representing the observed genotypes of all cells, α is the FPR, β is the FNR, and *k* is the parameter of the *k*-Dollo parsimony model. The algorithm starts with the tree $T_{1}$ where only the root node presents in the tree and others are free nodes, and terminates if no free nodes are available or the score metric *s* is less than a predefined threshold κ.

1: **function** buildMutationTree(*D*, α, β, *k*, λ, κ)
2: **Initialize:**
3: *M*← number of columns of matrix *D*;
4: ζ ← *M*(*k* + 1) + 1;
5: $T_{1}^{*}\leftarrow$ vector (0,-1,-1,.,-1) that has ξ elements;
6: $T^{*}\leftarrow T_{1}^{*}$, $L^{*}\leftarrow l\left(D|T^{*},\alpha ,\beta \right)$;
7: **for** *t* = 2 to ζ **do**
8: generate neighborhoods of the precursor tree $T_{t-1}^{*}$ as $\left\{T_{t}\right\}$;
9: calculate score of each tree $T_{p}^{c}\in \left\{T_{t}\right\}$ as $s\left(T_{p}^{c}\right)=\lambda {\bar{\pi }}_{p}+\left(1-\lambda \right){\tilde{\pi }}_{p}$;
10: $T_{t}^{*}$ ← ${\arg\max}_{T_{p}^{c}}s\left(T_{p}^{c}\right)$;
11: **if** $s\left(T_{t}^{*}\right)<\kappa$ **then**
12: break;
13: **end** **if**
14: **if** $l\left(D|T_{t}^{*},\alpha ,\beta \right)>L^{*}$ **then**
15: $L^{*}\leftarrow l\left(D|T_{t}^{*},\alpha ,\beta \right)$;
16: $T^{*}\leftarrow T_{t}^{*}$;
17: **end** **if**
18: **end** **for**
19: **return** $T^{*}$;
20: **end** **function**

Based on the posterior distribution, we adopt the expected improvement (EI) (Mockus and Mockus, 1991) as the acquisition function to decide the next point *x*_*t*+1_ to evaluate by maximizing the EI function, i.e., *x*_*t*+1_ = argmax_*x*_EI_*t*_(*x*). The EI function EI_*t*_(*x*) measures the expected improvement over the current largest observed value $f_{t}^{*}=\max_{i\leq t}f\left(x_{i}\right)$ and is defined as:

(12) $$\begin{array}{l} {\text{EI}}_{t}\left(x\right):={𝔼}_{t}\left[{\left[f\left(x\right)-f_{t}^{*}\right]}^{+}\right] \end{array}$$

where *g*^+^ = max(*g*, 0) denotes the positive part, and *E*_*t*_ represents the expectation taken under the posterior distribution *f*(*x*)|*f*(*x*_1:*t*_). We repeatedly select a point to evaluate per time by following above presented procedure until the maximum number of steps is reached. Finally, the best values of (α, β) are returned as the solution. We integrate a previously developed BO package (Martinez-Cantin, 2014) into the framework of GRMT for parameter optimization.

### 2.4. Simulating Single-Cell Mutation Data

We simulate single-cell mutation data by first generating a mutation tree and then sampling single cells from the tree. The chronological order of mutations is emulated from a tree growing perspective based on the *k*-Dollo parsimony model. By following the simulation strategy employed in Jahn et al. (2016), single-cell mutation data are produced from the simulated mutation tree via cell attachment and mutation state inference. Each cell is randomly attached to one of the internal nodes of the mutation tree, and the mutation state of the cell is fetched through exploring the mutation evolution trajectory from the root to the attachment point. The generated mutation data is further tuned according to predefined FP, FN, doublet and missing rates. Detailed description of the simulation procedure is provided in Supplementary Methods.

### 2.5. Performance Metrics

To examine the performance of the proposed method in deciphering the underlying genotype matrix (GTM) and the chronological order of mutations, we compare GRMT to four state-of-the-art (SOTA) methods including SCITE, SiFit, SPhyR and RobustClone in various simulated datasets. infSCITE (Kuipers et al., 2017) and SiCloneFit (Zafar et al., 2019) are excluded from performance evaluation as we fail to get their results within an acceptable time frame. Two performance metrics adopted by Miura et al. (2018) and Chen et al. (2020) are employed to evaluate the consistency between the predicted and ground truth GTM: (1) the percentage of missing bases (MBs) correctly imputed and (2) the error rate of the GTM. The evaluations are performed with doublet samples excluded. To evaluate the construction errors of mutation trees, two metrics including CASet and DISC proposed by DiNardo et al. (2020) are utilized to measure distances between the recovered mutation tree and the ground truth. Specifically, we use CASet_∩_ and DISC_∩_ distances calculated using common mutations of the input trees, and each input tree is preprocessed to aggregate the mutations of which the chronological order is unknown by using the following strategy: (1) if an internal node *p* has only one child node *c* and no cells are attached to *p*, the chronological order of the mutations represented by *p* and *c* is unknown, therefore we aggregate *p* and *c* into a single internal node; and (2) this procedure is repeated until no internal nodes meet the condition. An example is illustrated in Supplementary Figure 1. The performance of mutation tree construction of GRMT, SCITE, and SPhyR are compared. The specific formulations for the evaluation metrics are provided in Supplementary Methods.

### 2.6. Simulated and Real Data

We build 6 simulated datasets (denoted by D1-D6) under different scenarios defined by several controlling factors: number of cells *N*, number of mutations *M*, FPR α, FNR β, missing rate η, doublet rate ρ and parameter *k* of the *k*-Dollo parsimony model. Each dataset is generated by changing at most two of the factors while keeping the remaining fixed. The default values are set to *N* = 200, *M* = 200, α = 0.01, β = 0.2, η = 0.1, ρ = 0.1, and *k* = 0, unless indicated otherwise. The specific settings for each dataset are as follows: β ∈ {0.1, 0.2, 0.3} for D1, η ∈ {0.1, 0.2, 0.3} for D2, *N* ∈ {100, 500, 1,000} for D3, *M* ∈ {100, 500, 1,000} for D4, *M* ∈ {100, 200} and *k* = 1 for D5, ρ = 0 and β ∈ (0.05, 0.3) for D6. In addition, 50 replicates of mutation trees are simulated per pair (*M*, *k*) for datasets D1-D5, and 100 mutation trees are generated for dataset D6. This results in 1100 GTMs for comprehensively evaluating the performance of GRMT. We also obtain real SCS data of a metastatic colorectal cancer (Leung et al., 2017) and a high grade serous ovarian cancer (McPherson et al., 2016; Roth et al., 2016) to further examine the effectiveness of GRMT.

### 2.7. Evaluations

We first apply GRMT and other methods to simulated datasets D1-D5 generated under various conditions. The FPR and FNR required as the inputs of each method are set to the ground truth values. For SCITE and SiFit, the number of restarts and length of each MCMC chain are set to 3 and 200,000, respectively, and all other parameters use the default values. We adopt the default settings as documented in SPhyR and RobustClone. The hyper-parameters are configured as λ = 0.7 and κ = 1 for GRMT on both simulated and real datasets. The performance metrics quantifying the quality of recovered GTM and mutation tree are measured to make a comparison between different methods. We then assess the ability of GRMT in accurately estimating the FNR on simulated dataset D6.

## 3. Results

### 3.1. Systematic Evaluation on Simulated Data

#### 3.1.1. The Effect of FN Errors

**Figure 2:**
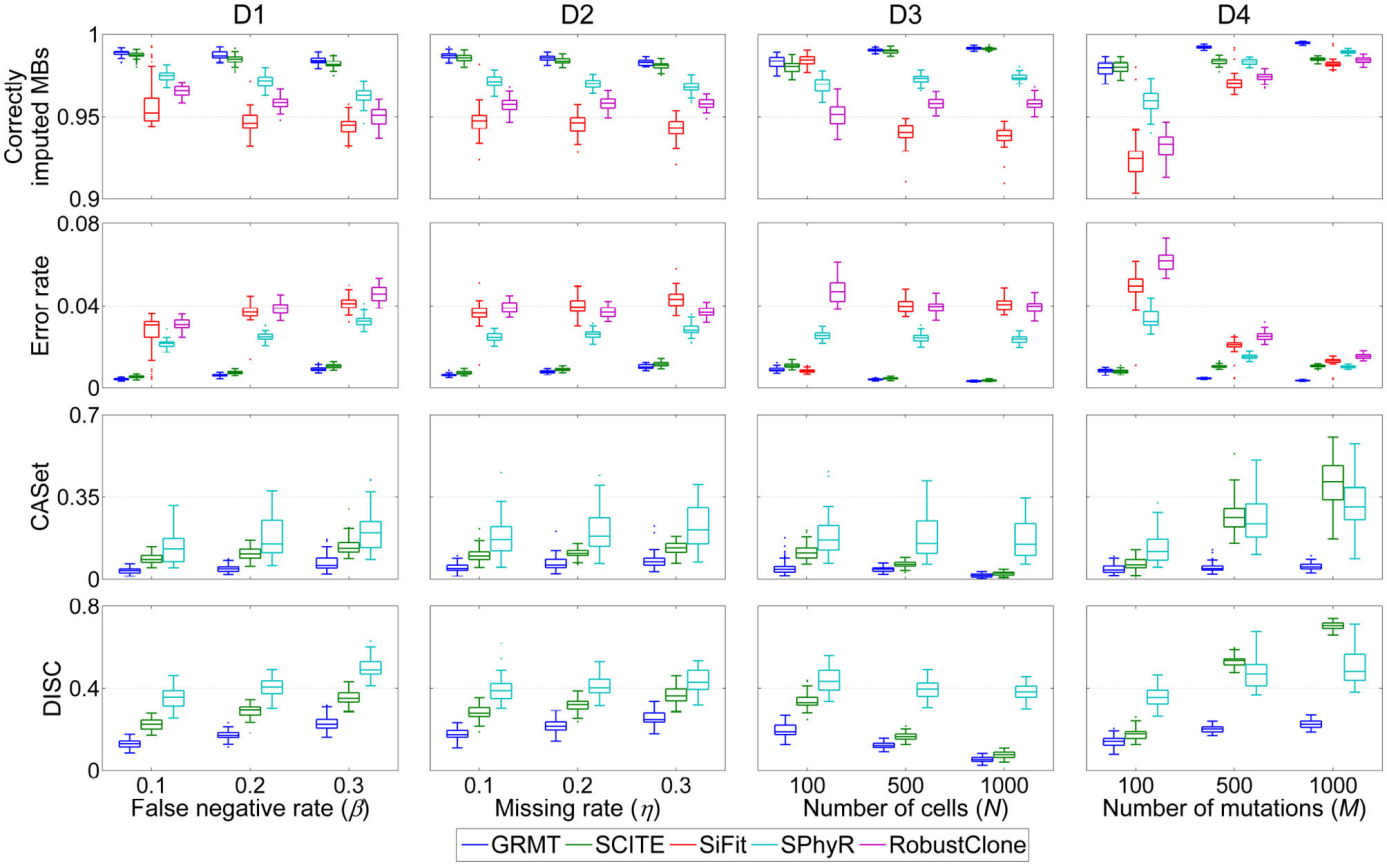
Performance comparison results of different methods in inferring GTM and tumor tree on simulated datasets. Four performance metrics including proportion of correctly imputed MBs, error rate of recovered mutation data, CASet and DISC distances measuring quality of reconstructed tumor tree are calculated with respect to each of four controlling factors. These factors include false negative rate (β), missing rate (η), number of cells (*N*) and number of mutations (*M*).

We first evaluate the effect of FN errors on GTM and mutation tree inferences, and make a comparison between different methods on the dataset D1. As shown in Figure 2, the distributions of four performance metrics are analyzed under different β values in {0.1, 0.2, 0.3}. It is observed that the ratio of correctly imputed MBs consistently decreases with the β for all methods. For instance, SiFit yields 95.22, 94.6, and 94.48% median accuracies when β changes from 0.1 to 0.3. SCITE outperforms other existing methods on all evaluations with median accuracy ranging from 98.19 to 98.75%. Compared to the competitors, our method achieves high robustness against β value. It delivers a median accuracy of 98.92% at β = 0.1, and results in 0.51% accuracy loss when β increases to 0.3. Further comparison results between the predicted GTM and ground truth indicate the proposed method has enhanced ability to precisely correct erroneous mutation calls. GRMT presents better results than SiFit, SPhyR and RobustClone by reducing the error rate by a large margin across all investigated β values, and also exhibits advantage over SCITE. In addition, analysis of CASet and DISC distances between the reconstructed and ground truth mutation trees gives similar comparison results. Since SPhyR primarily aims to infer subclonal trees, it results in low-resolution profiles of the mutation trees that present relatively low similarity to the ground truth. SCITE achieves more robust results than SPhyR, the median value of CASet distance increases from 0.083 at β = 0.1 to 0.13 at β = 0.3, and the DISC metric is also better than that of SPhyR. By comparison, our method generates more consistent mutation tree structure across all testing conditions. For instance, the median CASet distance is as low as 0.036 at β = 0.1 and 0.058 at β = 0.3, and the corresponding DISC distances are also smaller than those of SCITE (0.129 vs. 0.226 and 0.226 vs. 0.351). The comparison results suggest our method is able to cope with FN errors in SCS data.

#### 3.1.2. The Effect of Missing Data

We then evaluate the effect of missing data on the GTM and mutation tree reasoning on the dataset D2. Similar evaluation strategy is applied to benchmarking tests under different η values in {0.1, 0.2, 0.3}. Compared to the results on evaluation of β, SiFit, SPhyR and RobustClone show smaller variances in recovering GTMs with respect to η as shown in Figure 2, implying their higher robustness against missing rate than FNR. SCITE performs best among the existing methods by effectively eliminating the effects of missing data, and delivers the lowest variance in each metric with respect to η. By comparison, our method produces results comparable to SICTE by correctly interpolating at least 98.3% missing entries and reducing error rate to below 0.01 on most tests. For reconstructing mutation tree, SPhyR, SCITE and GRMT show similar performance as in correcting FN errors on dataset D1, and our method still gets better results than the competitors. For instance, the median values of CASet distances derived from GRMT, SCITE and SPhyR are 0.073, 0.132, and 0.208 at η = 0.3, respectively, and the corresponding DISC distances are 0.248, 0.363, and 0.428. These results indicate our method has superior performance in imputing missing data.

#### 3.1.3. The Effect of Number of Cells and Mutations

We proceed to assess the effectiveness of GRMT on large SCS datasets D3 and D4. As expected, GRMT and SCITE yield improved results when more cells are employed to recover the GTM and mutation tree. For instance, our method reduces the median error rate from 0.89% at *N* = 100 to 0.32% at *N* = 1000, representing an elevated capability when compared to SCITE (corresponding values are 1.09 and 0.36%). The performance of SiFit tends to deteriorate when the number of cells increases, which may result from under-convergence of the model caused by the high computational complexity in inferring lineage relationship among a large number of cells. SPhyR outperforms SiFit and RobustClone at larger values of *N*, and shows better robustness to the change in number of cells.

The number of mutations also acts as one of the main factors that heavily affect the results of existing methods. With more mutations incorporated into the analysis, the mutational difference between cells get enhanced and the effects of technical errors are substantially attenuated, enabling an elevated accuracy of SiFit, SPhyR and RobustClone as depicted in Figure 2. SCITE performs comparably to GRMT at *M* = 100, but shows degraded capability at larger values of *M* due to low efficiency of the MCMC scheme in deciphering evolutionary history of a large number of mutations. Generally, our method presents best results under different test conditions, and gains higher robustness to the change in number of mutations. For instance, the errors in GTM are corrected to account for a small median percent of 0.35 at *M* = 1,000, and the CASet distance slightly increases from 0.039 at *M* = 100 to 0.051 at *M* = 1,000.

#### 3.1.4. Evaluation on Mutation Loss Data

**Figure 3:**
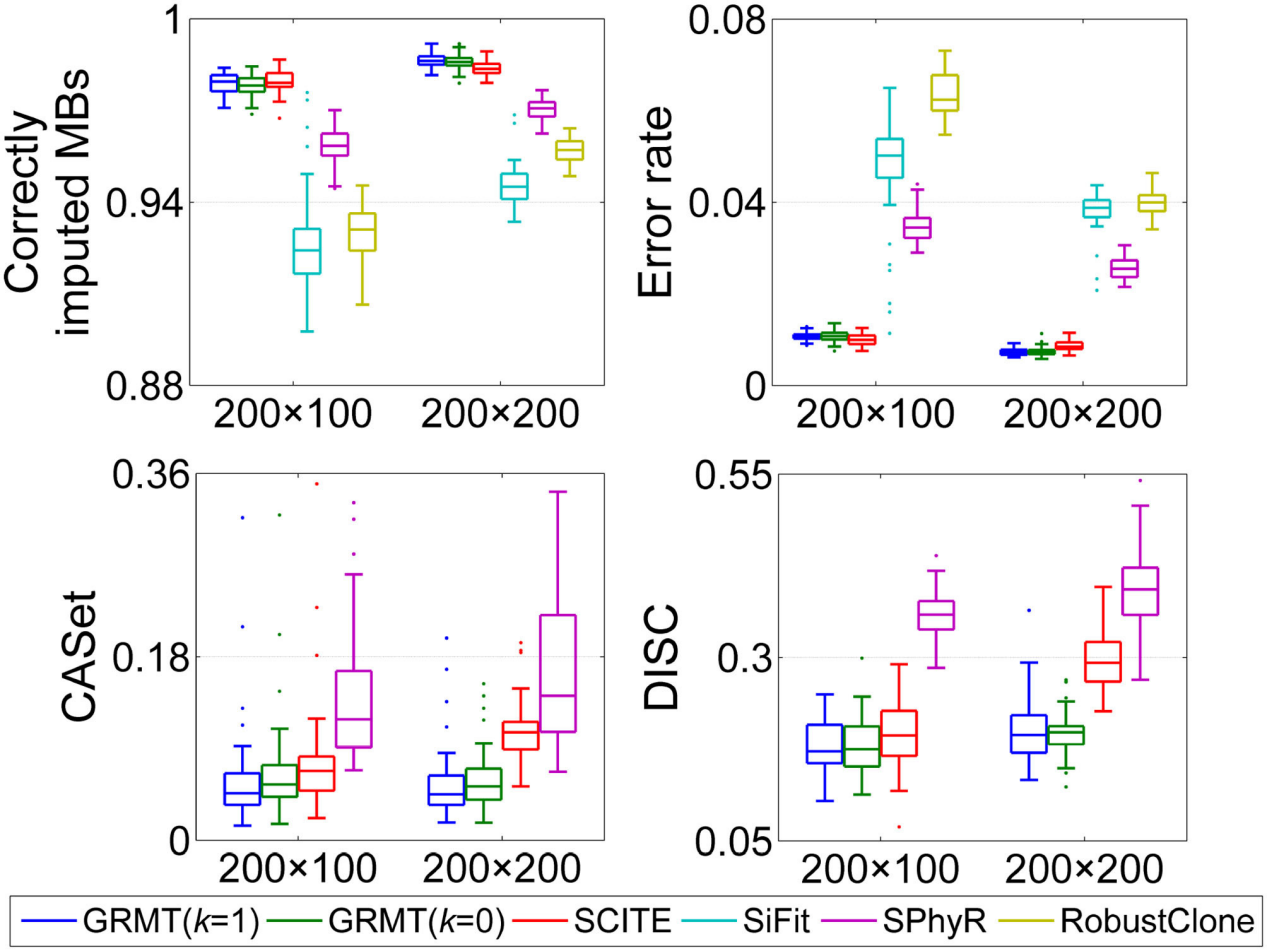
Comparison results on mutation loss data. The performance of GRMT gets improved when modeling loss of mutation with *k* = 1. The evaluations are performed on 200 × 100 and 200 × 200 GTMs.

To examine the ability of reasoning tumor evolutionary history involved with mutation loss, we apply GRMT to dataset D5. We run GRMT under two ways, i.e., *k* = 0 and *k* = 1, and compare the resulting metrics with the SOTAs. As shown in Figure 3, with *k* = 1 GRMT shows better metrics when compared to the results with *k* = 0, and yields generally better inferences than the ISM based methods. For instance, the median values of the CASet distance derived from GRMT (*k* = 1), GRMT (*k* = 0), SCITE and SPhyR on 200 × 100 GTMs are 0.046, 0.055, 0.068, and 0.119, respectively, and the corresponding values on 200 × 200 GTMs are 0.045, 0.053, 0.106, and 0.142. The overall performance of GRMT with either *k* = 0 or *k* = 1 is better than the competitors, especially in deducing the underlying mutation trees.

#### 3.1.5. Estimating the False Negative Rate

**Figure 4:**
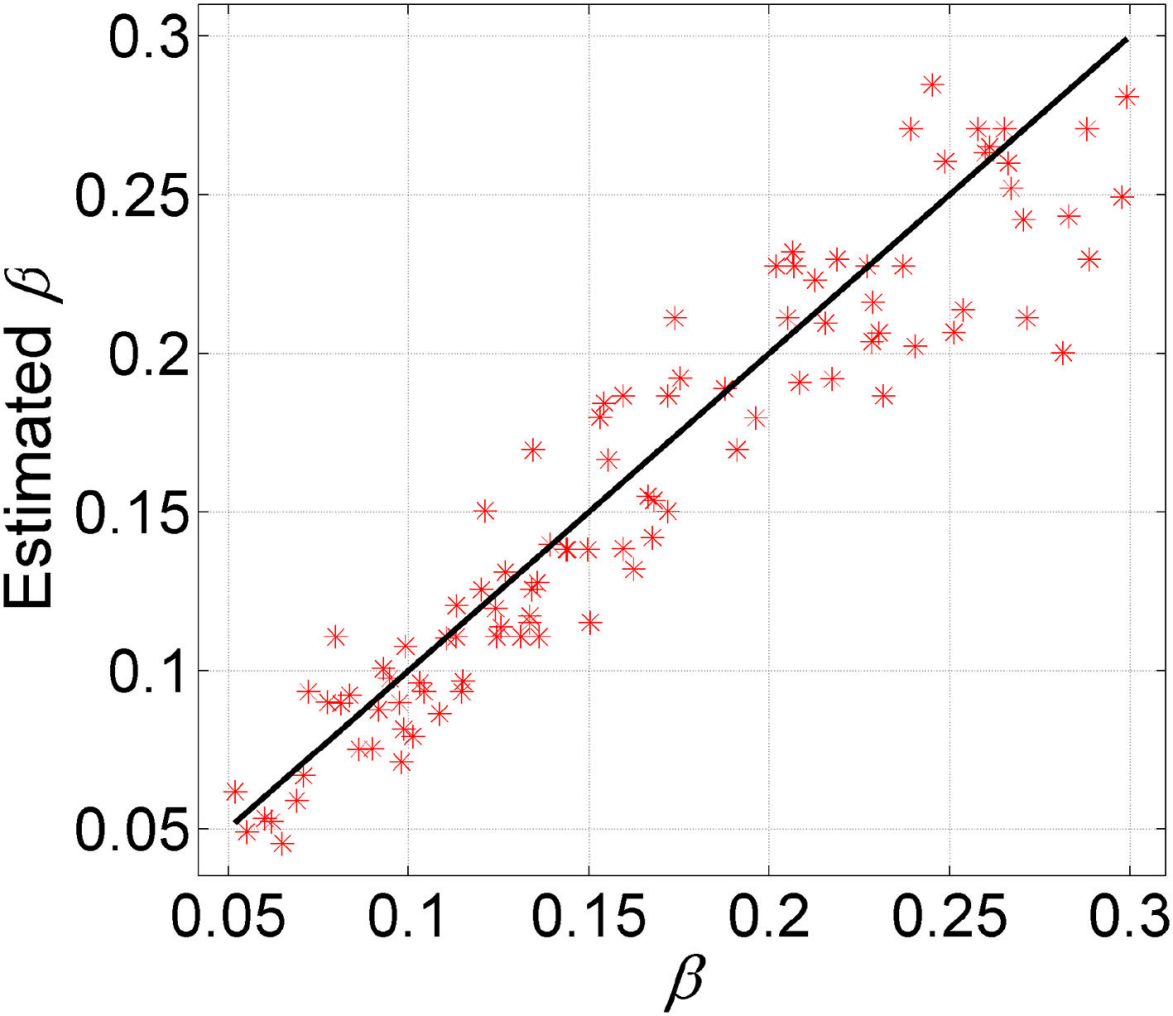
Comparison between estimated FNR and the ground truth. GRMT accurately estiamtes the FNR with high correlation to the ground truth that generates the data.

To examine the ability of GRMT in estimating the FNR, we apply GRMT to dataset D6. For the BO algorithm, the number of initial sampling points and iterations is set to 50 and 15, respectively. The results depicted in Figure 4 imply the FNR is accurately estimated by GRMT with high correlation (correlation coefficient of 0.94 and *p*-value of 1.88 × 10^−48^) to the ground truth that generates the data, suggesting GRMT performs well in inferring FNR from the highly disturbed mutation data.

#### 3.1.6. The Effect of Hyper-Parameters

**Figure 5:**
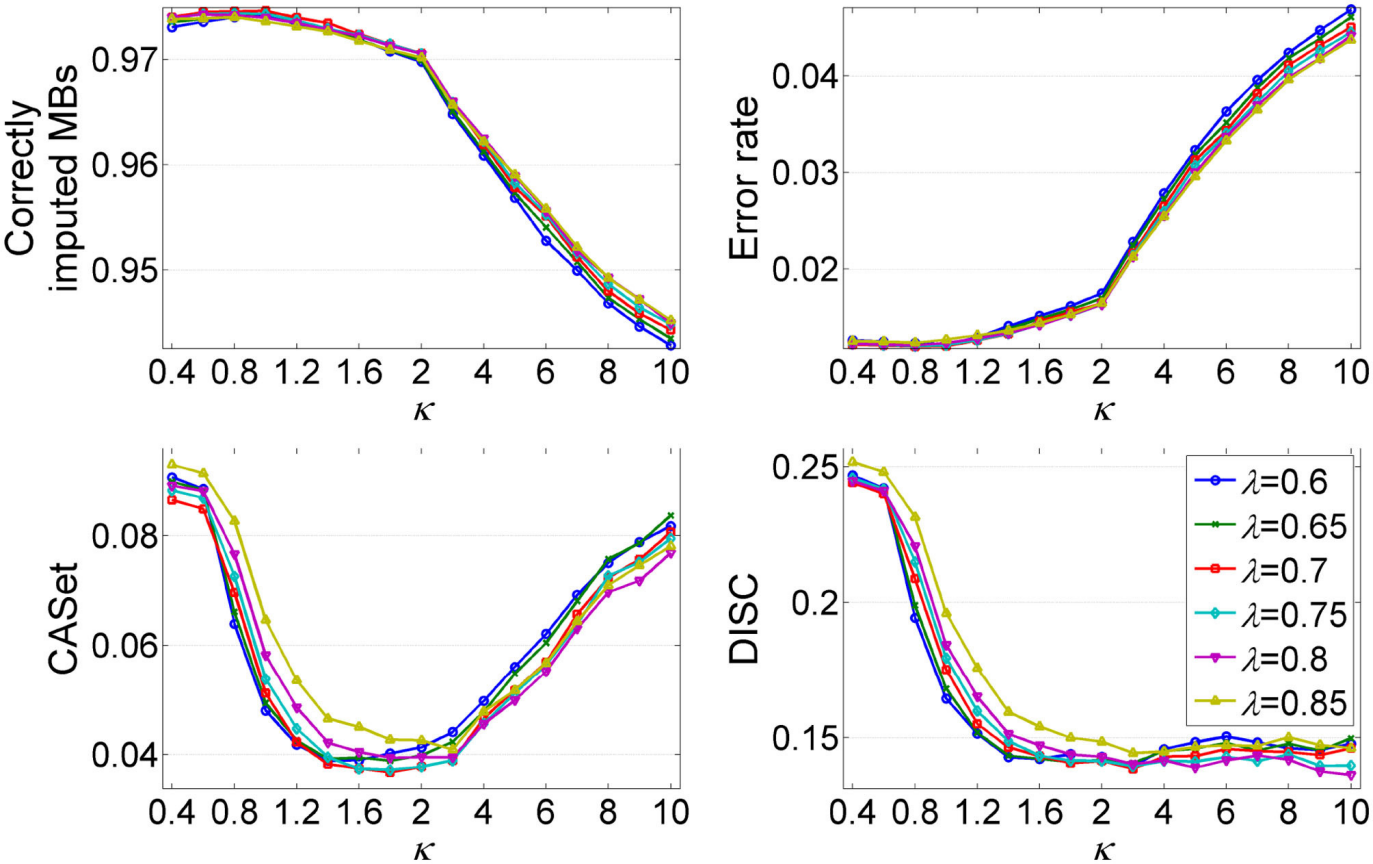
Analysis of the effects of hyper-parameters λ and κ on inference results of GRMT. The λ changes from 0.6 to 0.85, and κ varies from 0.4 to 2. Four performance metrics are measured under different (λ, κ) pairs.

The parameters λ and κ are two important hyper-parameters in GRMT, and control the expansion direction and depth of the mutation tree, respectively. To investigate the effect of λ and κ on inference results, we compare the performance metrics under different combinations of λ and κ values. The λ changes from 0.6 to 0.85, and κ varies from 0.4 to 10. The produced dataset consists of 50 100 × 100 GTMs per (λ, κ) pair, for which the mean value of each metric is measured. As shown in Figure 5, each metric changes obviously with κ, and shows similar patterns across different λ values. The recovered GTM yields the best results when λ = 0.7 and κ ≤ 1, while presents dropped accuracy with κ > 1 across all λ values. Since larger κ values will result in early stopping of the tree expansion, the cells near to ends of the branches cannot be correctly attached to right positions of the mutation tree, which contributes to the elevated error rate of the GTM. Inversely, small κ value encourages depth expansion of the tree, thus gives a relatively high probability of introducing unexpected edges that violate to the ground truth. As expected, the CASet distance approximately follows a V shaped relationship with κ and the minimum is reached near κ = 1.8. The inferred mutation tree exhibits decreased DISC distance with the baseline tree when κ increases from 0.4 to 3, and shows little changes in DISC distance at κ > 3. Taken together, (λ = 0.7, κ = 1) is an appropriate choice that provides a good tradeoff among different metrics.

In addition, we examine the performance of GRMT and the competitors on various simulated mutation trees with different levels of structural complexity. The structural complexity of mutation tree is controlled by parameter γ as described in Supplementary Methods. The results in Supplementary Figures 2, 3 demonstrate our method is more accurate in handling mutation trees with complex structure. More details on the evaluations can be found in Supplementary Results.

#### 3.1.7. Runtime Performance

**Figure 6:**
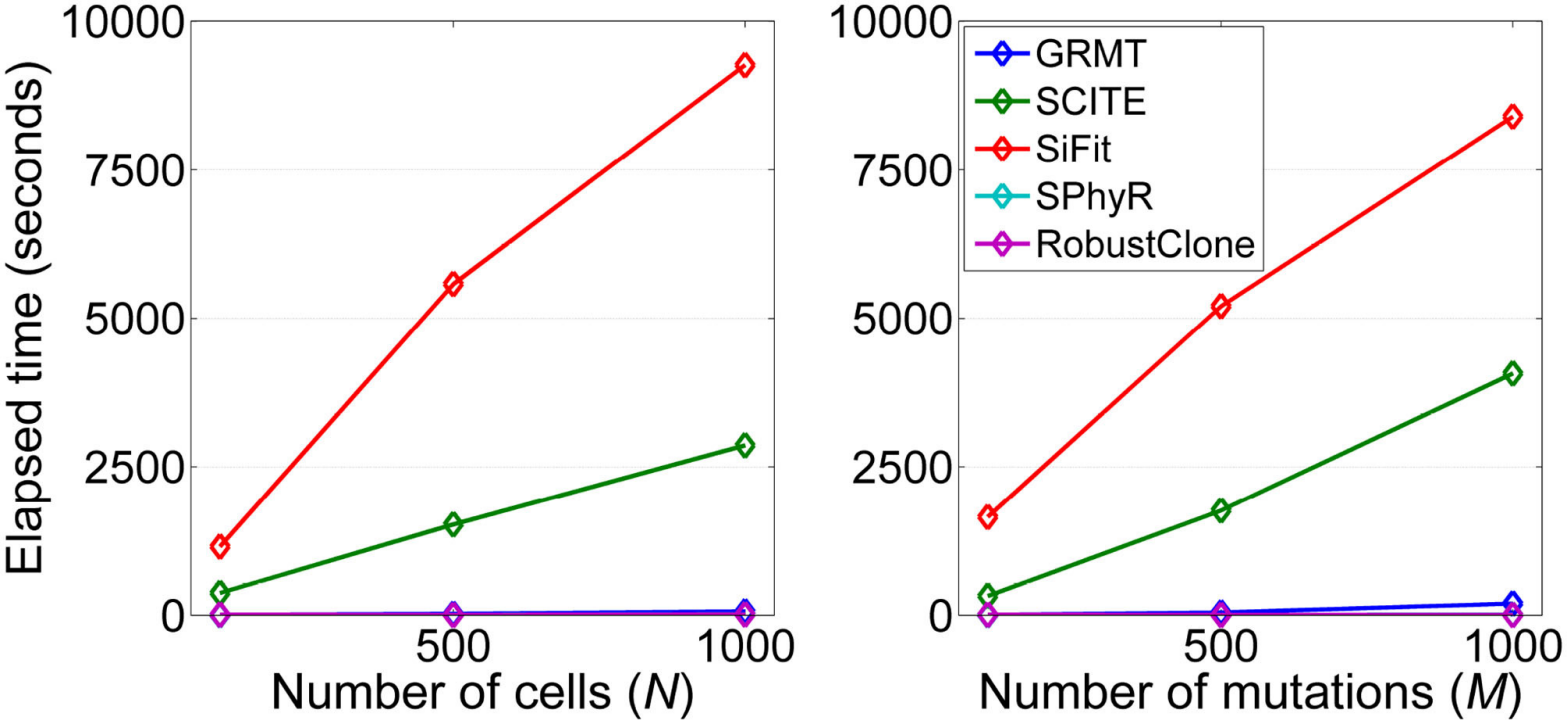
Computational efficiency of the investigated methods. The running time with respect to each of the two controlling factors including number of cells (*N*) and number of mutations (*M*) are measured.

We analyze the computational efficiency of the investigated methods on datasets D3 and D4. Figure 6 shows the results of elapsed time of each method respect to the number of cells and mutations. It is observed that GRMT presents comparable performance with SPhyR and RobustClone, and is significantly more efficient than SCITE and SiFit. For instance, GRMT requires average 65 s to process 1000 × 200 mutation data, while SCITE and SiFit need 2,864 and 9,265 s, respectively. To further examine the efficiency of GRMT on larger datasets, we simulate a dataset consisting of 2,000 × 500 GTMs with α = 0.01, β = 0.2, η = 0.1, and ρ = 0.1. The calculated performance metrics shown in Supplementary Figure 4 indicate our method outperforms the competitors in all performance metrics. The average per-sample processing time of GRMT, SCITE and SiFit are 7, 270, and 1,974 min, respectively, suggesting GRMT scales well to large datasets.

### 3.2. Reconstructing Evolutionary Histories From Real Data

#### 3.2.1. Applying GRMT to Metastatic Colorectal Cancer Dataset

We use GRMT to recover the evolutionary history of a metastatic colorectal cancer from patient CRC1 (Leung et al., 2017). The dataset contains 178 single cells sampled from primary and metastatic cancer tissues. Variant calling finds 16 single-nucleotide variants (SNVs) from the cells, resulting in a 178 × 16 binary mutation matrix with approximately 6.7% missing rate. The FPR and FNR are estimated as α = 1.52 and β = 7.89%, respectively.

We first test GRMT under the ISM assumption, i.e., *k* = 0. With the fixed error rates (α = 1.52%, β = 7.89%), GRMT achieves a log-likelihood of –396.11 and the inferred mutation tree is depicted in Supplementary Figure 5. Previous studies (Zafar et al., 2017; El-Kebir, 2018) have reported identification of three subclones with somatic mutations as well as the population without mutations, we mark each population with a specific color. Most of the diploid cells are attached to the root of the mutation tree (marked in gray), implying they are normal stromal cells. The tumor is initiated by the mutation in the *KRAS* oncogene, and develops with the subsequent mutations in the *APC* and *TP53* tumor suppressor genes. These mutations delineate the first subclone (marked in blue) consisting mostly of diploid cells. Subsequent mutations acquired in genes like the *ROBO2* tumor suppressor gene and *CCNE1* oncogene result in emergence of the second subclone (marked in green) that contains mostly primary aneuploid cells. Further accumulation of mutations in *ZNF521, TRRAP*, and *RBFOX1* result in the metastatic clade that forms the third subclone (marked in red), and mark the point of tumor dissemination to the liver. The subclone consists of metastatic cells only, and most of cells acquire additional mutations in *EYS* and *GATA1* genes. By learning the error rates from the data, our method achieves a higher log-likelihood of –351.96, and estimates the parameters as α = 1.04 and β = 7.90%, respectively. The reconstructed mutation tree in Supplementary Figure 6 suggests approximately one-third of the primary aneuploid cells have the *TPM4* mutation and constitute a separate clade of the second subclone.

**Figure 7:**
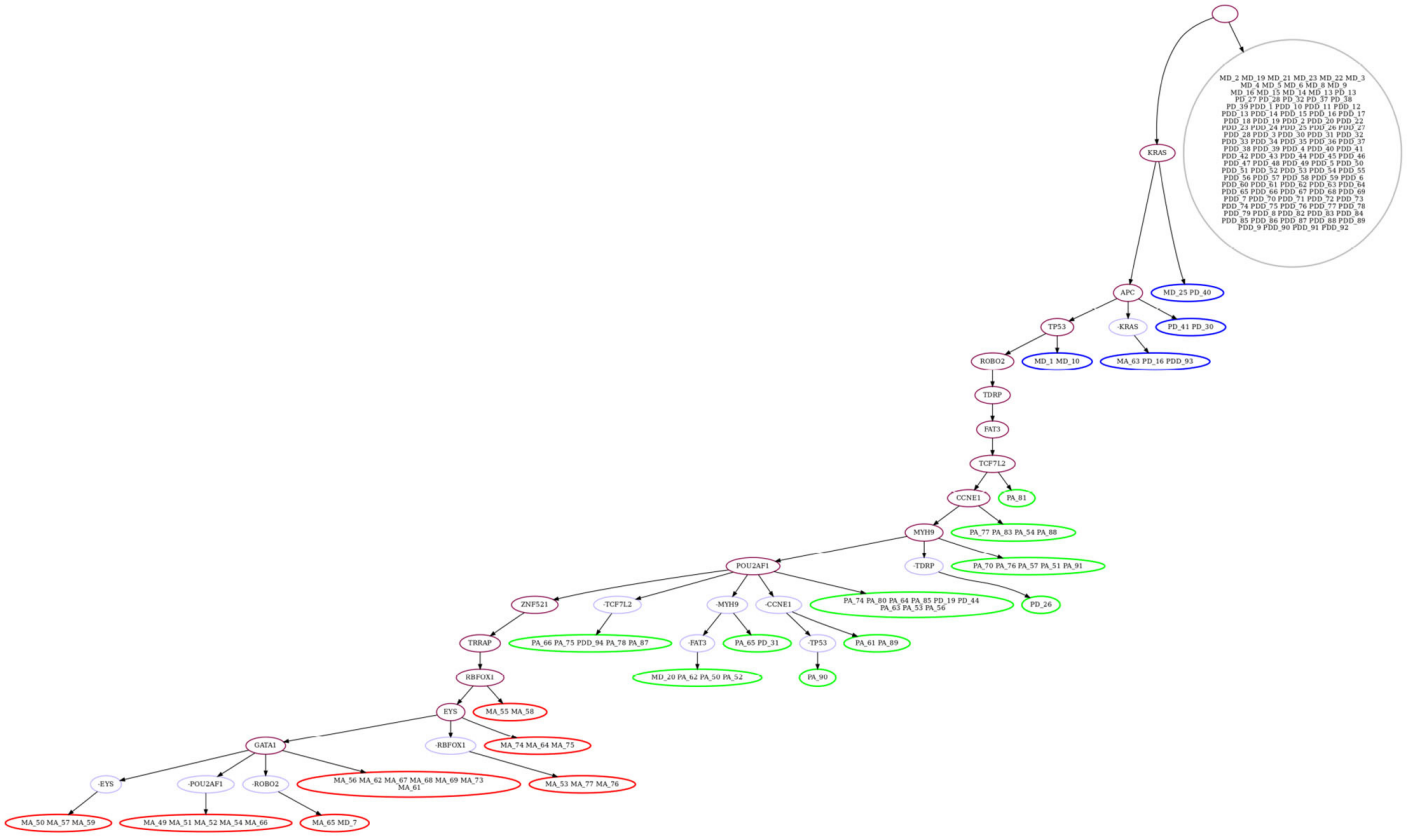
Mutation tree inferred by GRMT with α = 1.52%, β = 7.89% and *k* = 1 on metastatic colorectal cancer dataset. GRMT yields a improved log-likelihood of –314.06. The tree contains 11 mutation losses (prefixed by “”), and loss of mutation mainly occur in the primary and metastatic aneuploid cells. The normal population (marked in gray) consists of diploid cells, the first subclone (marked in blue) consists mostly of diploid cells, the second subclone (marked in green) contains mostly primary aneuploid cells, and the third subclone (marked in red) consists of metastatic cells only.

We then evaluate GRMT by allowing each mutation to be lost at most one time, i.e., *k* = 1. Under the fixed error rates (α = 1.52%, β = 7.89%), GRMT gets a elevated log-likelihood of -314.06. The recovered mutation tree in Figure 7 implies there are 11 mutation losses, and loss of mutation events mainly occur in the primary and metastatic aneuploid cells. For instance, the mutation in *POU2AF1* acquired in the primary aneuploid cells is lost in 5 metastatic cells. The evolutionary branches associated with loss of mutation enhance the resolution of clonal architecture by refining the inner-clone cell diversity. We further analyze the results derived from learning the error rates from the data. GRMT yields a significantly improved log-likelihood of –282.09 with α and β estimated as 1.07 and 5.72%, respectively. The inferred mutation tree in Supplementary Figure 7 contains 10 mutation losses, and also suggests the mutation in *TPM4* only occurs in the primary aneuploid cells.

SCITE is also applied on this dataset with α = 1.52% and β = 7.89%. SCITE outputs a mutation tree (shown in Supplementary Figure 8) with a log-likelihood of –337.71. It also identifies the three subclones and the normal population following similar evolutionary patterns as the ones inferred by GRMT.

#### 3.2.2. Applying GRMT to High Grade Serous Ovarian Cancer Dataset

We further examine GRMT on a high grade serous ovarian cancer (HGSOC) dataset (McPherson et al., 2016; Roth et al., 2016). The original HGSOC dataset consists of 420 cells and 43 mutations. Following the previously adopted strategy (Roth et al., 2016), we exclude low-quality cells that show high rates of mutation missing, resulting in a 392 × 43 GTM with 8.6% missing rate. Due to the FPR and FNR are unknown for this dataset, we use GRMT to jointly infer the error rates and the mutation tree with *k* ∈ {0, 1}. With *k* = 0, our method estimates the α and β as 4.39% and 34.1% with a log-likelihood of -7320.5. The reconstructed mutation tree in Supplementary Figure 9 provides a high-resolution landscape of the clonal architecture, and suggests multiple highly divergent subclones exists in the tumor, which is similar to the previously reported result (Roth et al., 2016). The tumor is initiated by the mutation in the *TP53* tumor suppressor gene, then evolves into two branches one of which forms a separate clade after accumulating mutations in genes like *BRCA1* tumor suppressor gene and *CENPI* oncogene (marked in cyan). Another branch further splits into two clades after acquiring the mutation in *YTHDF3*. One of the clades (marked in red) consists of 113 cells of which 112 cells derive from the left ovary, while another clade excludes the LOv1 cells. This clade is characterized by an initial set of mutations that present in non-LOv1 cells (marked in green), and further extended by the mutations absent in LOv2 cells (marked in blue). By allowing loss of the mutations with *k* = 1, GRMT achieves a significantly improved log-likelihood of -6676.88 by learning the error rates as α = 2.73% and β = 29.7%. The inferred mutation tree (Supplementary Figure 10) has 42 mutation losses and yields a higher resolution indication of the clonal architecture refined by mutation loss events.

We also apply SCITE on this dataset with α = 4.39% and β = 34.1%. SCITE infers a mutation tree (shown in Supplementary Figure 11) with the log-likelihood of -7312.88. It also identifies divergent subclones that evolve through similar patterns as the ones found by GRMT.

## 4. Discussion

With the ability of delivering the genetic diversity at single cell resolution, SCS is particularly useful to decipher intra-tumor heterogeneity in cancer. In this study, we develop a new computational method GRMT for accurate and efficient reconstruction of mutation tree based on SCS data. As loss of mutation is the dominant factor that contributes to the homoplasy of SNVs in cancer (El-Kebir, 2018), GRMT leverages the *k*-Dollo parsimony model to impose a restriction on mutation evolution that each mutation can be gained once and lost at most *k* times. We elaborate a generative framework to reconstruct mutation tree from scratch by iteratively introducing new node to the tree per mutation event. To best explain the observed error-prone mutation data, BO based parameter tuning is employed to infer the maximum likelihood estimation of the error rates. Compared to the existing tools, the advantages of GRMT lie in three aspects: (1) the generative model enables accurate and fast inference of chronological order of mutations; (2) the BO algorithm is more efficient than MCMC based approaches in estimating the FPR and FNR; (3) the good tradeoff between computational efficiency and inference accuracy makes GRMT scales very well to large datasets. We perform extensive evaluation of GRMT on simulated and real datasets to demonstrate its superior performance in profiling clonal architecture from SCS data.

One limitation of GRMT lies in the fact that it does not explicitly model doublet events, thus may suffer from degraded performance when applied to datasets with high doublet rates, and we plan to elaborate on this issue in the future. In addition, there are several potential directions for future research to improve the performance of GRMT. First, the errors in genotypes is non-uniformly distributed across the cells, thus exploiting genotype uncertainty available from SNV callers can result in an improved inference (Wu, 2020). This suggests a Bayesian framework that utilizes this information may yield more accurate results. Second, inclusion of other data sources may also be a feasible direction to refine the results solely derived from SCS data. A previous study (Malikic et al., 2019) proposes to infer tumor evolutionary history from combined bulk and SCS data, where the fitness of the model to bulk data is also optimized. Finally, copy number information inferred from sequencing data (Yuan et al., 2019; Satas et al., 2020) may be useful to restrict the mutations that have undergone losses, thus shrinks the search space of candidate mutation trees.

## Data Availability Statement

The original contributions presented in the study are included in the article/Supplementary Material, further inquiries can be directed to the corresponding author/s.

## Author Contributions

ZY and FD conceived the study. ZY designed the methods and implemented the GRMT algorithm and wrote the first draft of the manuscript. HL and XT analyzed the data. All authors contributed to manuscript revision, read, and approved the submitted version.

## Conflict of Interest

The authors declare that the research was conducted in the absence of any commercial or financial relationships that could be construed as a potential conflict of interest.
