# Supplementary material for: GRMT: Generative Reconstruction of Mutation Tree From Scratch Using Single-Cell Sequencing Data
Source: Front Genet. 2021 Jun 4;12:692964. doi: 10.3389/fgene.2021.692964 (PMC8212059; doi:10.3389/fgene.2021.692964)
Supplement: Supplementary file 1 [file Data_Sheet_1.PDF]

GRMT: Generative reconstruction of mutation tree from scratch using single-cell sequencing data Supplementary Material

Zhenhua Yu *[1,2], Huidong Liu[1], Fang Du[1,2] and Xiaofen Tang[1,2]

[1] *School of Information Engineering, Ningxia University*
[2] *Collaborative Innovation Center for Ningxia Big Data and Artificial Intelligence Co-founded by Ningxia Municipality and Ministry of Education, Ningxia University*

Contents

1 Supplementary Methods **2**
1.1 Simulating single-cell mutation data 2
1.2 Formulations for performance metrics 2

2 Supplementary Results **3**
2.1 Results on simulated datasets generated with different γ values 3

3 Supplementary Figures **4**

*Corresponding author: zhyu@nxu.edu.cn

1 Supplementary Methods

1.1 Simulating single-cell mutation data

We simulate single-cell mutation data by first generating a mutation tree and then sampling single cells from the leaves of the tree. The chronological order of mutations is emulated from a tree growing perspective by following the k-Dollo parsimony model. Given the number of mutations M to mimic, we first generate $M(k+1)$ isolated tree nodes with labels $(1+, 1-, 1-, \ldots, 1-, 2+, 2-, 2-, \ldots, M+, M-, M-, \ldots, M-)$, where symbols "+" and "-" denote gain and loss of a mutation respectively. We then initialize the mutation tree to only contain the root node suggesting no mutations. The tree is iteratively extended by introducing a new node per time from the isolated nodes based on a predefined node weights W that provides a probability distribution to sample nodes. We give higher weights to the nodes that labeled with "+" to encourage mutation accumulation in the early stage of tree growing. The attachment position of new node in the mutation tree is sampled from internal nodes by leveraging the weights of all internal nodes. Formally, we formulate the weight of the i-th internal node as $\exp(\gamma/(c_i+1))$, where c_i represents the number of child nodes of the i-th internal node and $\gamma > 0$ is a scaling factor (set to 5 in all simulated experiments). The probability that the i-th internal node is selected as the attachment point is given by $p_i = \frac{\exp(\gamma/(c_i+1))}{\sum_j \exp(\gamma/(c_j+1))}$. The values of γ affect the growing direction of the mutation tree, i.e. higher values of γ give more preference to linear evolution and potentially yield deeper mutation trees, while lower values of γ encourage more branches and can produce wider mutation trees. Such relationship is illustrated in Fig. S12 that gives a comparison between the generated mutation trees under different γ values. The growth of the tree continues until all nodes labeled with "+" are attached to the tree. Single cells are then sampled from the generated mutation tree, and the mutation data is further tuned according to predefined false positive, false negative, doublet and missing rates.

1.2 Formulations for performance metrics

We use two metrics to evaluate the quality of recovered genotype matrix (GTM): the percentage of correctly imputed missing bases (MBs) and the error rate of the recovered GTM. Suppose the input, output, and ground truth GTMs are D, B and B^* respectively, correctly imputed MB is a missing entry in D that has equal value in B and B^*. The percentage of correctly imputed MBs is the percent of correctly imputed MBs in all missing entries in D, and the error rate is the proportion of the entries in B that have different value in B^*. In addition, we adopt two distance metrics, i.e. CASet and DISC distances proposed by a previous study (DiNardo *et al.*, 2020), to measure the reconstruction errors of mutation trees. The distance metrics are defined based on the topology of the trees and the labels present in the vertices (mutations) of the trees, we employ the same formulations as in (DiNardo *et al.*, 2020) to calculate the two distance metrics.

References

DiNardo, Z., Tomlinson, K., Ritz, A., and Oesper, L. (2020). Distance measures for tumor evolutionary trees. *Bioinformatics*, **36**(7), 2090–2097.

2 Supplementary Results

2.1 Results on simulated datasets generated with different γ values

To examine the effects of parameter γ on the performance of GRMT and the competitors, we compare the results on simulated datasets generated under different γ values in $\{0.1, 1, 10\}$. For each value of γ, the number of mutations is set to 200, and 30 mutation trees are generated. From each mutation tree, two GTMs with size of 200×200 and 500×200 are simulated. We then test all methods on these simulated data under the same parameter settings as on other simulated data. The measured performance metrics on 200×200 and 500×200 mutation data are shown in Figs. S2 and S3, respectively. The results demonstrate GRMT outperforms the existing methods on datasets with $\gamma = 0.1$ or $\gamma = 1$, and also shows comparable performance to SCITE in larger γ value ($\gamma = 10$), suggesting our method is more accurate in handling mutation trees with complex structure and SCITE has advantage in recovering mutation trees with linear structure.

3 Supplementary Figures

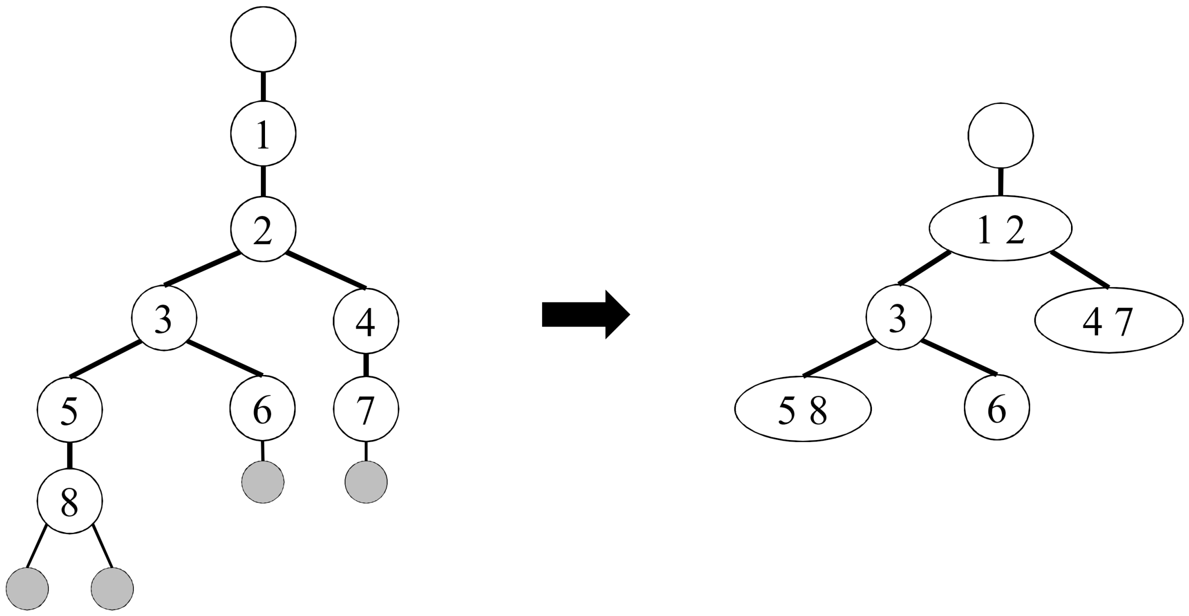

Figure S1: An illustration of mutation tree preprocessing before calculating tree distances. The left subfigure represents the original mutation tree where single cells are marked in gray, and the right subfigure shows resulting tree. If the chronological order of mutations represented by two connected nodes is unknown, the two nodes are aggregated into a single node. This procedure is repeated until no nodes meet the condition.

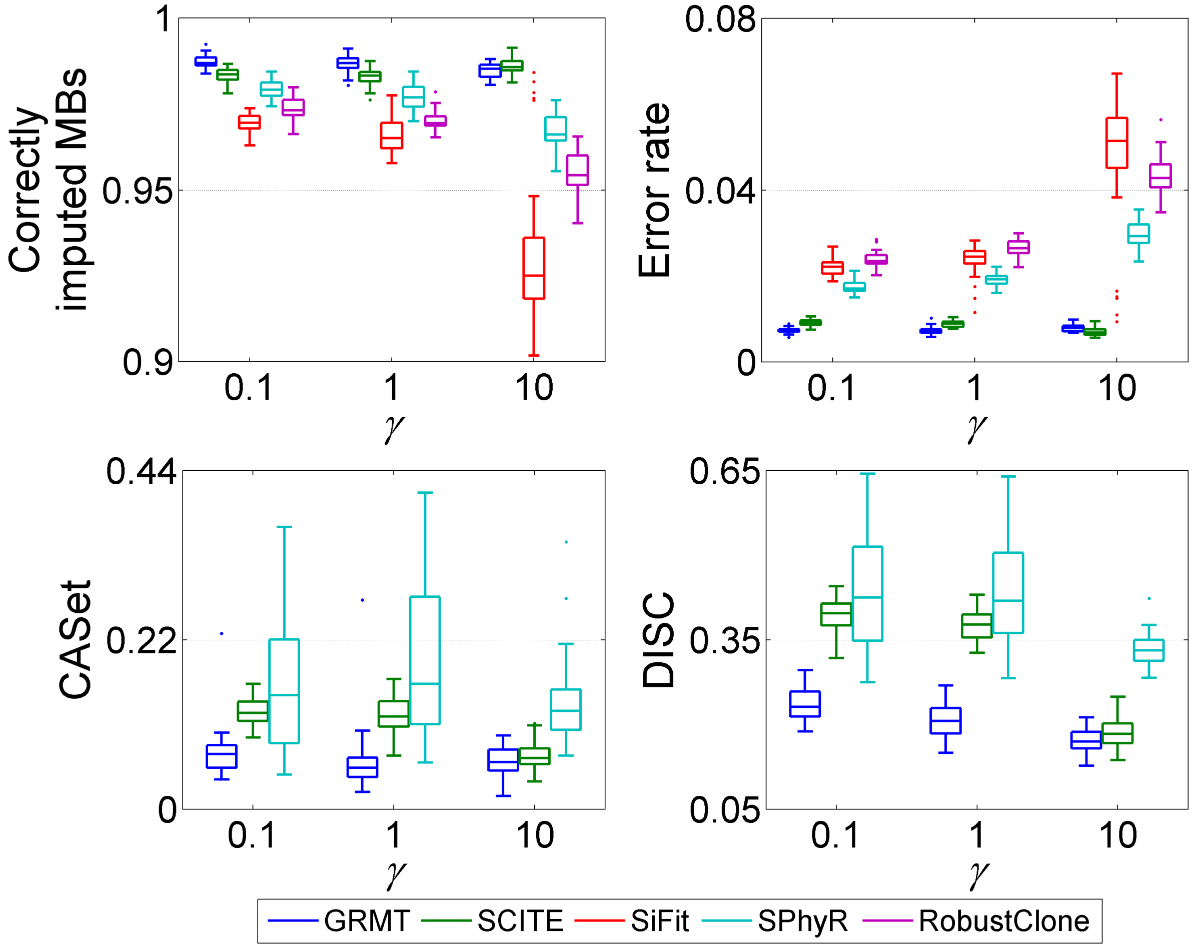

Figure S2: Performance comparison results of different methods on 200×200 simulated matrices with different γ values.

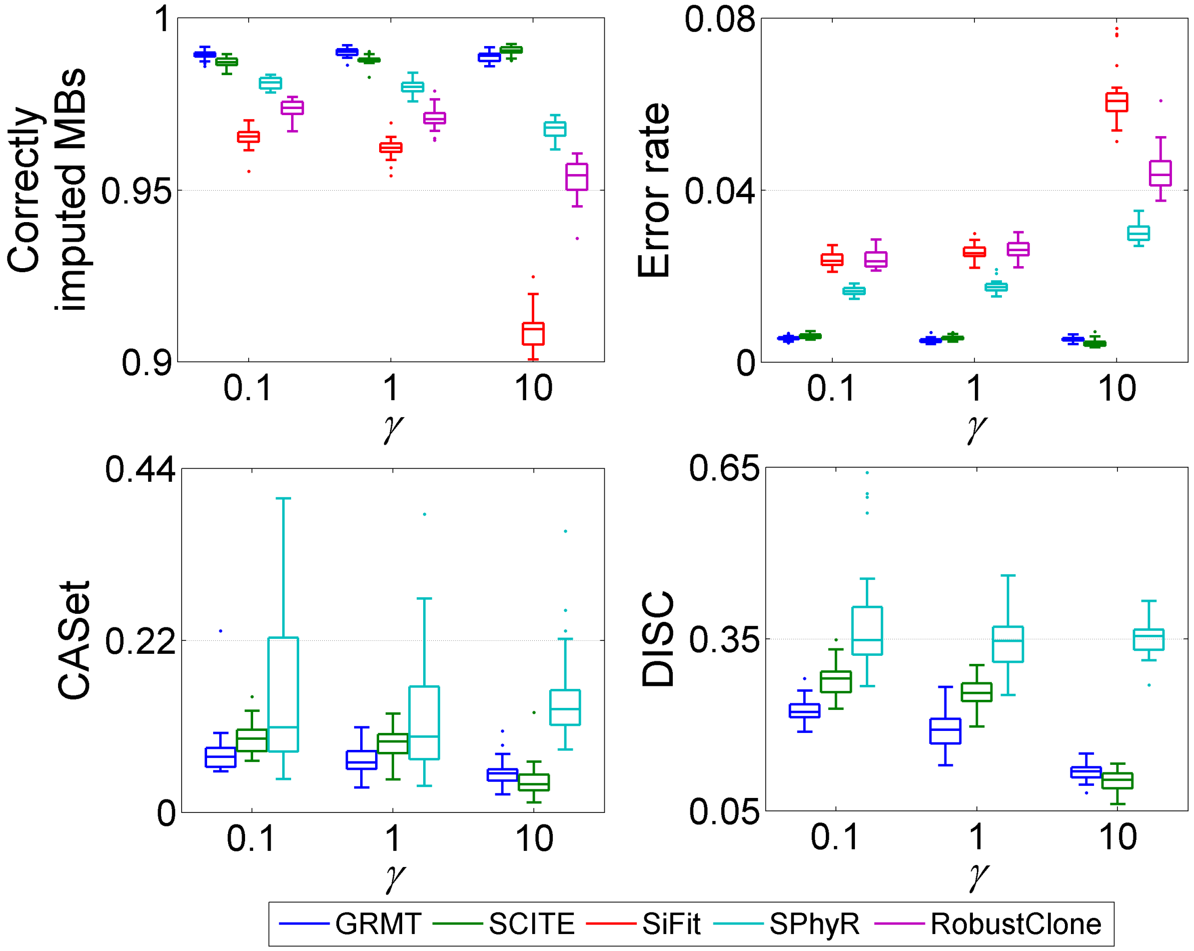

Figure S3: Performance comparison results of different methods on 500×200 simulated matrices with different γ values.

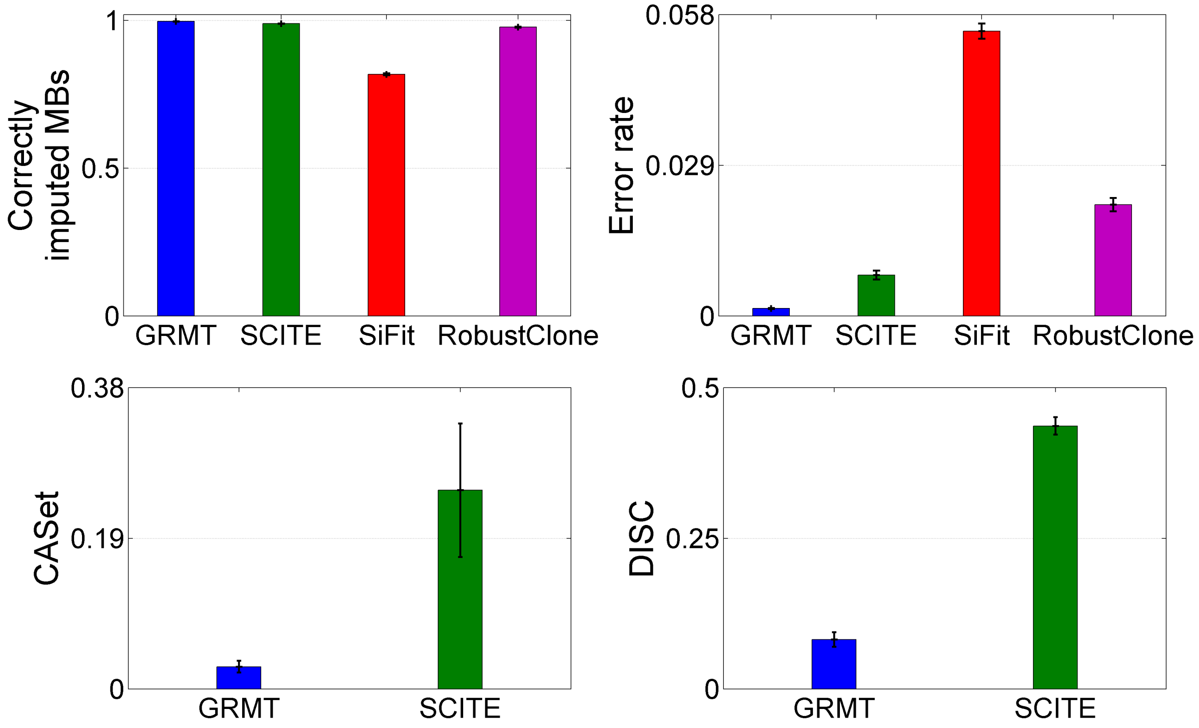

Figure S4: Performance comparison results of different methods on 2000×500 simulated matrices. As SPhyR reports a runtime error when dealing with the data, we exclude the comparison results of SPhyR.

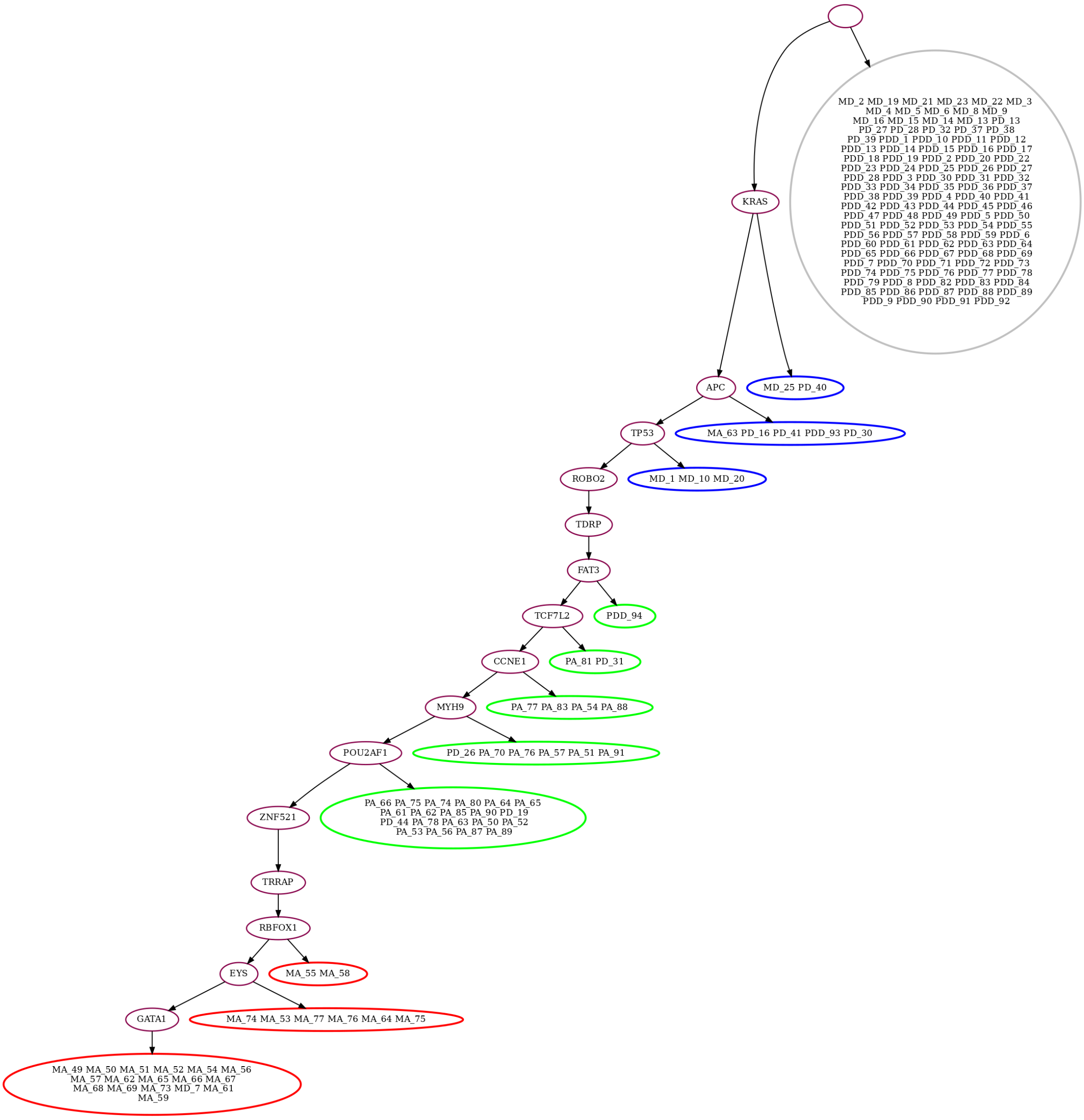

Figure S5: Mutation tree inferred by GRMT with $\alpha = 1.52\%$, $\beta = 7.89\%$ and $k = 0$ on metastatic colorectal cancer dataset. GRMT yields a log-likelihood of -396.11. Three subclones with somatic mutations as well as the population without mutations are identified. The normal population (marked in gray) consists of diploid cells, the first subclone (marked in blue) consists mostly of diploid cells, the second subclone (marked in green) contains mostly primary aneuploid cells, and the third subclone (marked in red) consists of metastatic cells only.

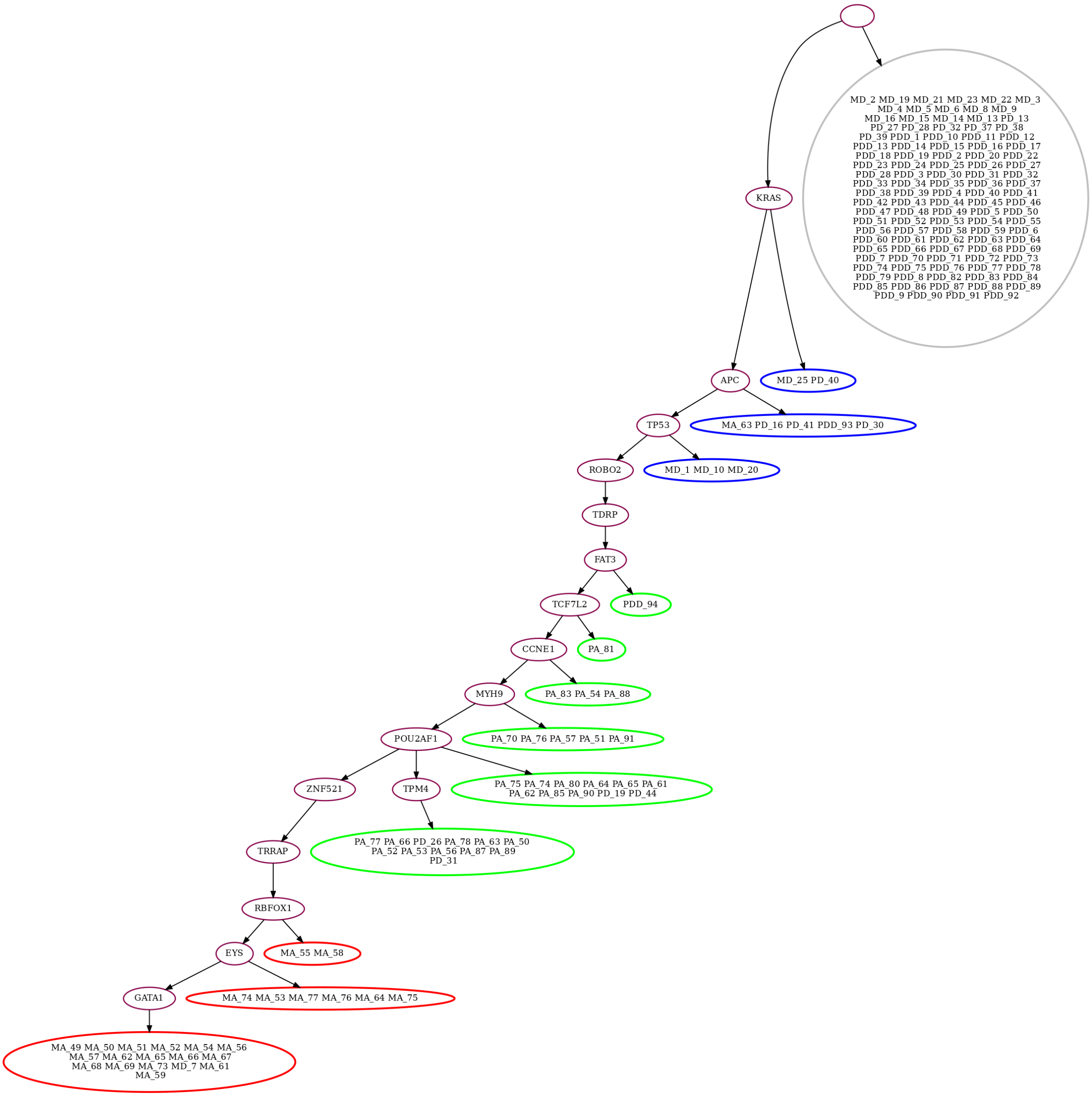

Figure S6: Mutation tree inferred by GRMT with α = 1.04%, β = 7.90% and $k = 0$ on metastatic colorectal cancer dataset. The FPR α and FNR β are estimated from the data, and a log-likelihood of -351.96 is achieved. GRMT still identifies three subclones with somatic mutations and the normal population.

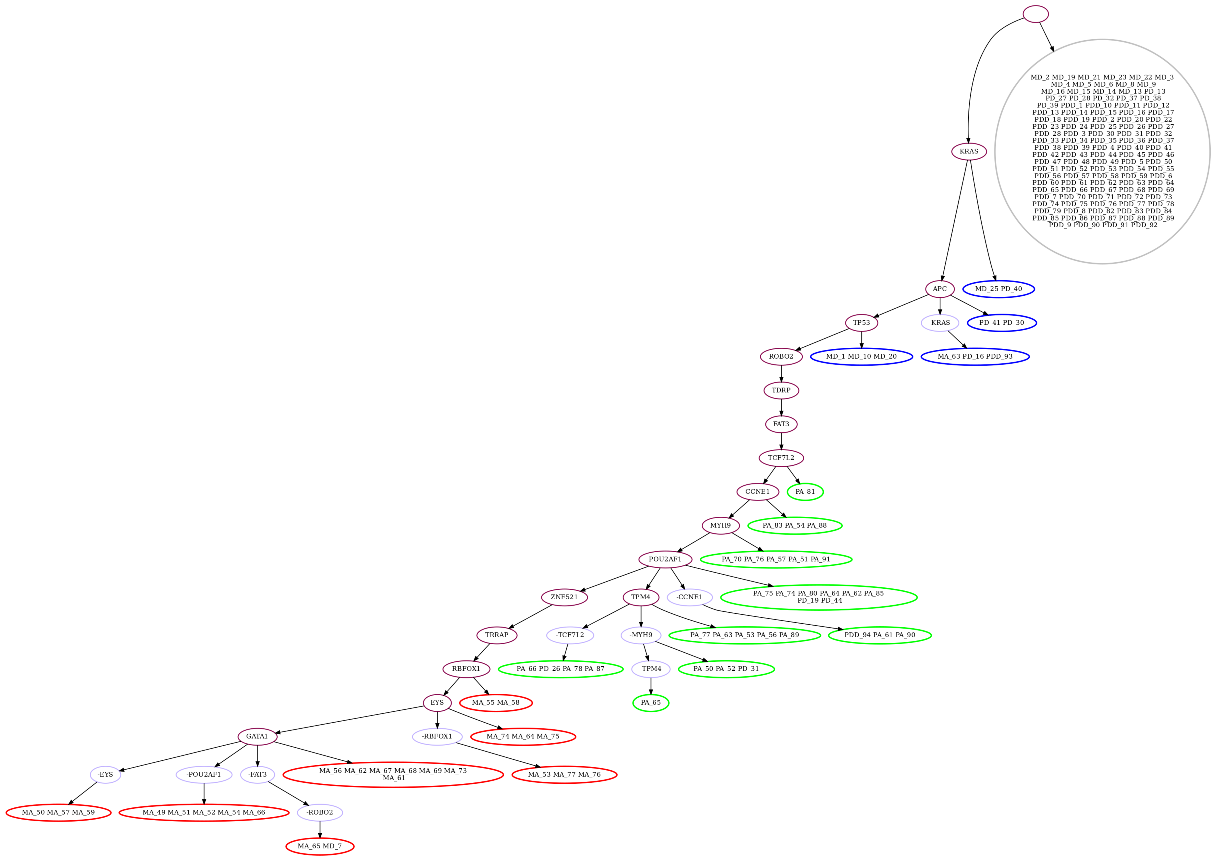

Figure S7: Mutation tree inferred by GRMT with $\alpha = 1.07\%$, $\beta = 5.72\%$ and $k = 1$ on metastatic colorectal cancer dataset. GRMT estimates the FPR α and FNR β as 1.07% and 5.72%, respectively, and yields a significantly improved log-likelihood of -282.09. The tree contains 10 mutation losses.

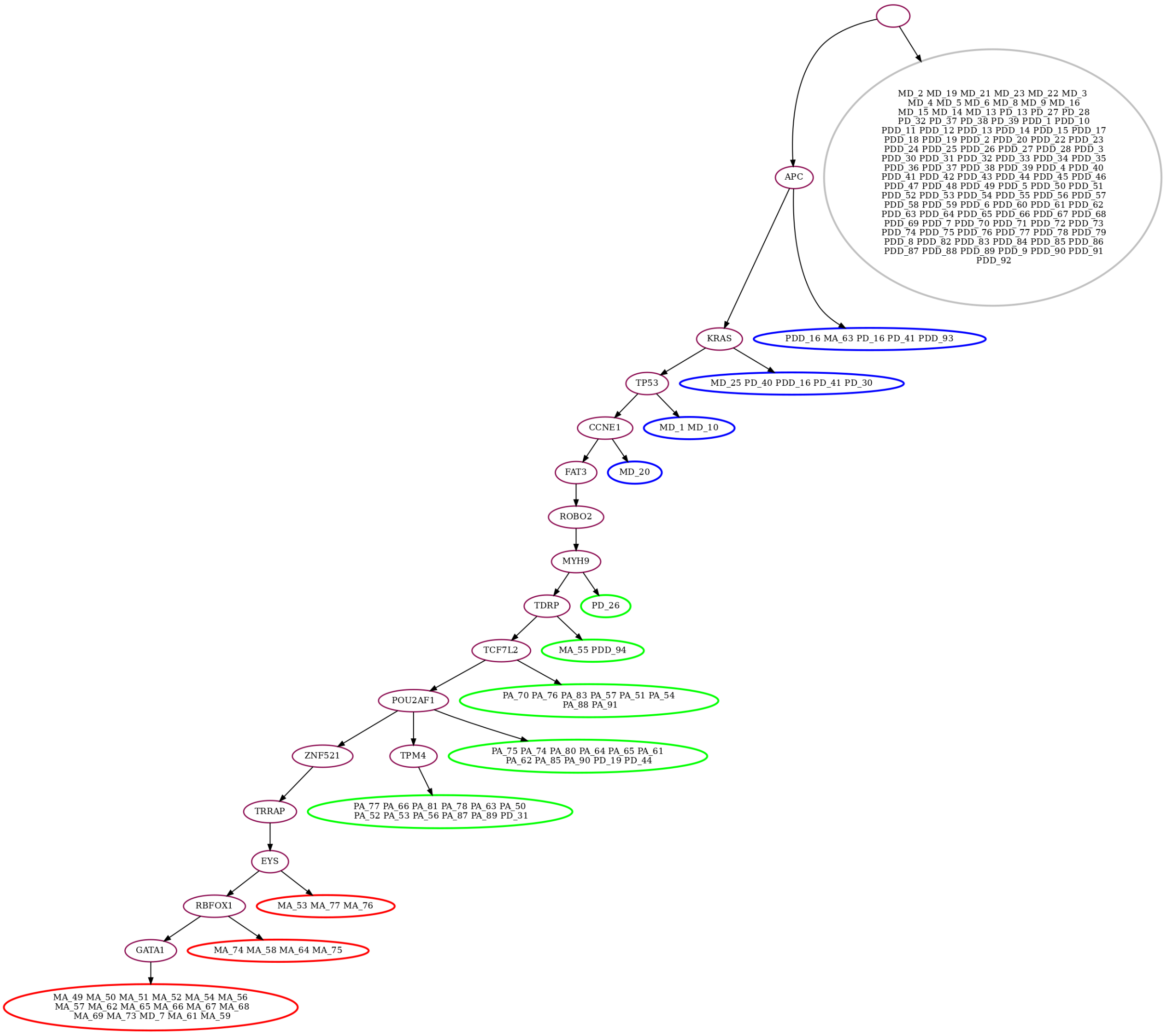

Figure S8: Mutation tree inferred by SCITE with $\alpha = 1.52\%$ and $\beta = 7.89\%$ on metastatic colorectal cancer dataset. SCITE achieves a log-likelihood of -337.71, and identifies similar clonal composition to that of GRMT.

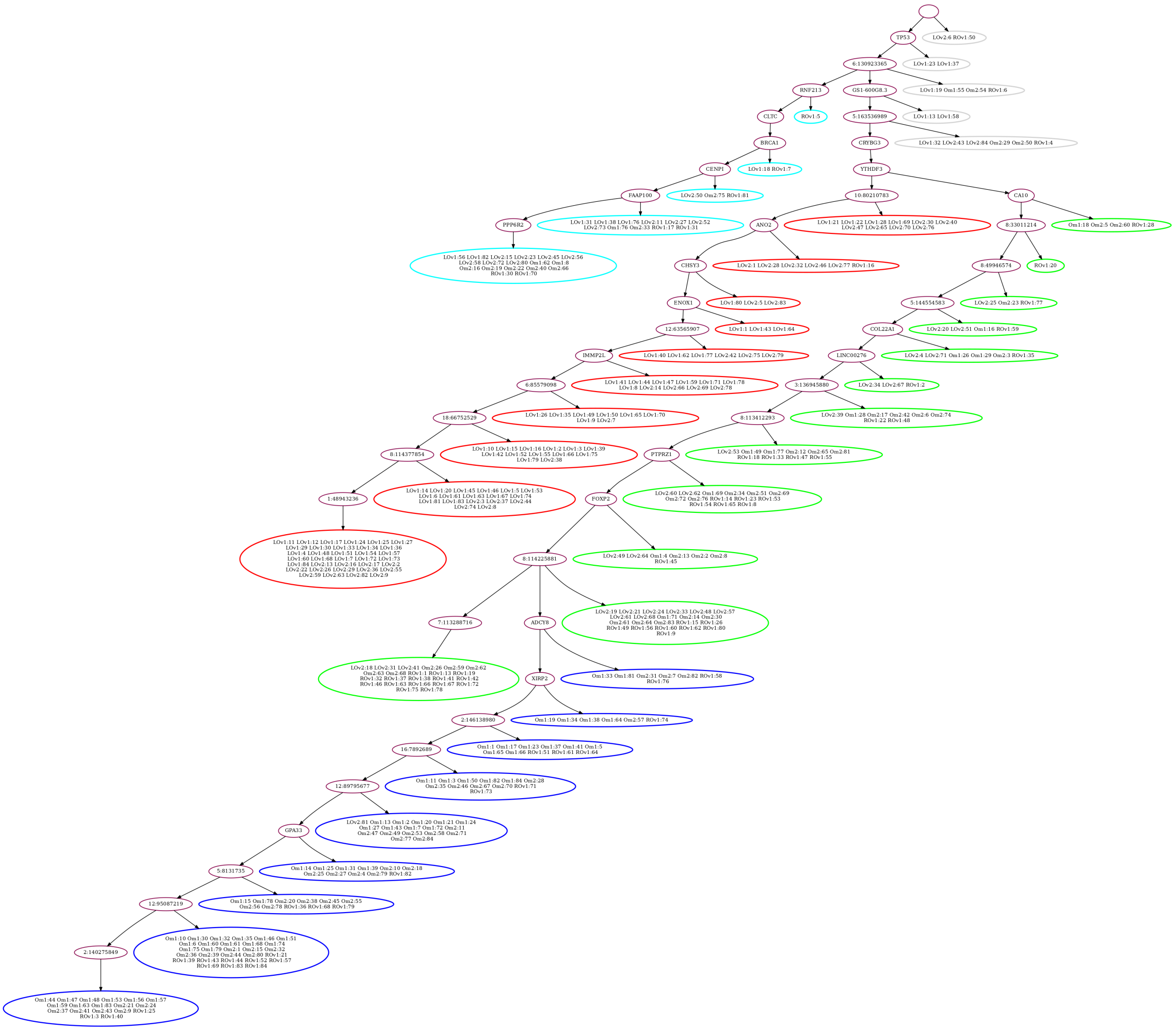

Figure S9: Mutation tree inferred by GRMT with $\alpha = 4.39\%$, $\beta = 34.1\%$ and $k = 0$ on high grade serous ovarian cancer dataset. GRMT estimates the FPR α and FNR β as 4.39% and 34.1%, respectively, and yields a log-likelihood of -7320.5. The results suggest the tumor is initiated by the mutation in the *TP53* tumor suppressor gene, then evolves into multiple highly divergent subclones.

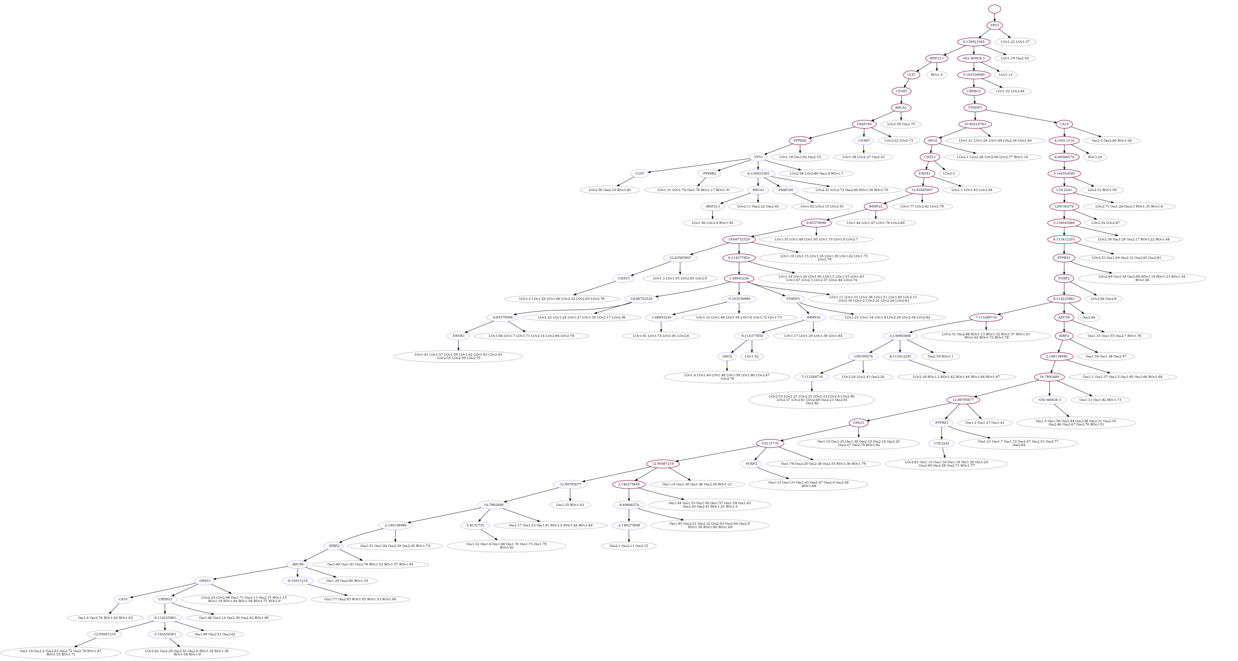

Figure S10: Mutation tree inferred by GRMT with $\alpha = 2.73\%$, $\beta = 29.7\%$ and $k = 1$ on high grade serous ovarian cancer dataset. GRMT estimates the FPR α and FNR β as 2.73% and 29.7%, respectively, and yields a larger log-likelihood of -6676.88. The results suggest there are 42 mutation losses (prefixed by "–").

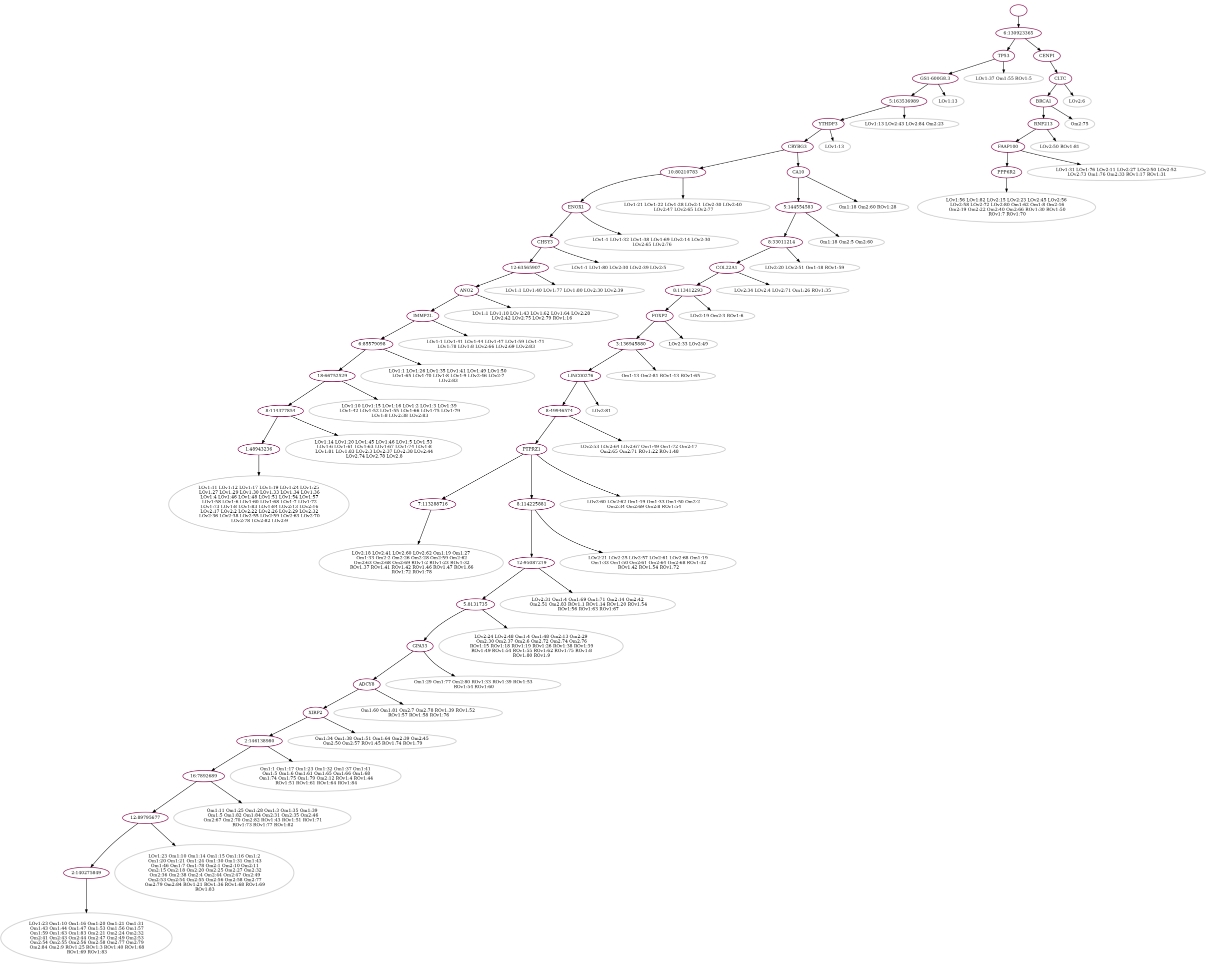

Figure S11: Mutation tree inferred by SCITE with $\alpha = 4.39\%$ and $\beta = 34.1\%$ on high grade serous ovarian cancer dataset. SCITE achieves a log-likelihood of -7312.88, and outputs a similar mutation tree to that of GRMT.

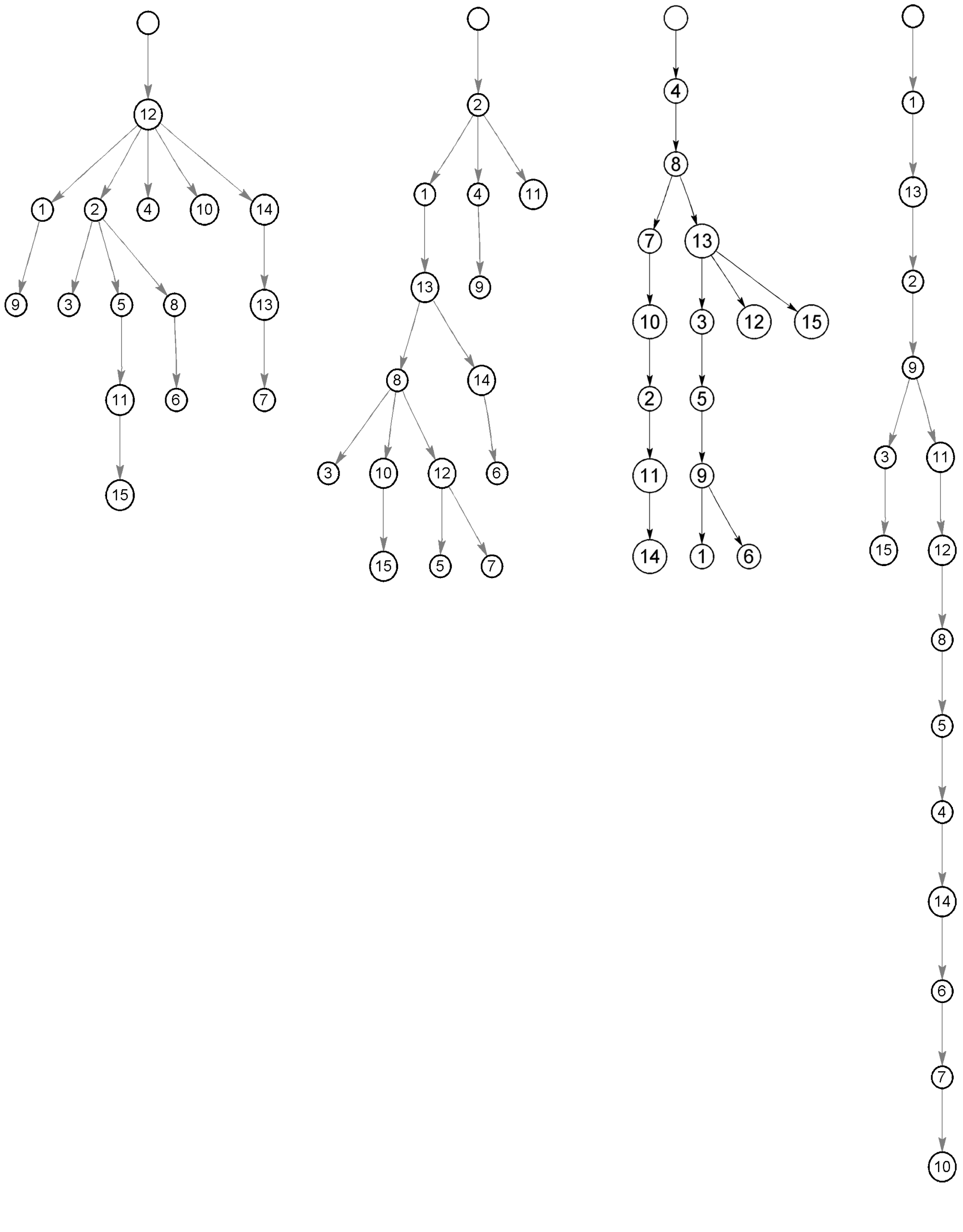

Figure S12: Illustration of the simulated mutation trees under different γ values. The γ values corresponding to the presented trees are 0.1, 1, 5 and 10, respectively.
